# Post-traumatic stress disorder in peacekeepers: a systematic literature review and meta-analysis

**DOI:** 10.1080/20008066.2024.2413735

**Published:** 2024-10-22

**Authors:** Laura Carmona, Cláudia Camilo, Vânia Sofia Carvalho, Maria José Chambel

**Affiliations:** a CicPsi, Faculdade de Psicologia da Universidade de Lisboa, Lisbon, Portugal; b CIS-Iscte, Iscte-IUL, Lisbon, Portugal

**Keywords:** PTSD, peacekeepers, peacekeeping, post-traumatic stress disorder, military, TEPT, personal de mantenimiento de la paz, mantenimiento de la paz, trastorno de estrés postraumático, militares

## Abstract

**Background:** In peacekeeping operations, soldiers are often exposed to the same traumatic factors as in conventional war and may also be subject to physical risks and psychological stressors associated with post-traumatic stress disorder (PTSD). According to the Conservation of Resources Theory (COR), PTSD stems from resource depletion and inadequate restoration.

**Objectives:** To discuss and meta-analyse PTSD-related factors among peacekeepers, based on the COR theory, framing them as resources or loss/threat of loss of resources.

**Methods:** A systematic literature search was performed with relevant keywords, 51 articles were reviewed and 21 of them meta-analysed.

**Results:** Factors mentioned in prior reviews, reinforced by ours, include: family/community and military support as resources; single marital status, female gender, serving in infantry, and longer time since deployment as lack of resources. Factors mentioned in prior reviews, confirmed by our meta-analysis, include: education, rank, and problem-focused coping as resources; negative perceptions about deployment, combat/trauma exposure, deployment stressors, and deployment duration as lack of resources. Factors overlooked in prior reviews include: age as a resource; negative life events, and negative social interactions as lack of resources. Comorbidities include: physical health problems, post-deployment impact on functioning, and post-deployment psychopathology (e.g., depression, substance use).

**Conclusions:** Significantly more individual than contextual factors were identified. While some factors inherent to missions (e.g., combat exposure, deployment stressors) cannot be mitigated, others are crucial to prevent peacekeepers’ PTSD (e.g., coping strategies, deployment duration, perceptions about deployment, social interactions, support during deployment) and to inform selection and monitoring by the Armed Forces (e.g., pre-, during and post-deployment psychopathology). However, the findings should be interpreted with caution due to limitations (e.g., publication bias, study heterogeneity) that may have affected the generalizability and strength of the recommendations.

## HIGHLIGHTS

- This study discusses and meta-analyses PTSD-related factors among peacekeepers, based on the Conservation of Resources Theory, framing them as resources or loss/threat of loss of resources.
- Identified resources include: family/community and military support; education, rank, problem-focused coping; age.
- Identified lack of resources include: single marital status, female gender, serving in infantry, longer time since deployment; negative perceptions about deployment, combat/trauma exposure, deployment stressors, deployment duration, negative life events, negative social interactions.

## Abstract (es)

**Antecedentes:** En las operaciones de mantenimiento de la paz, los soldados a menudo están expuestos a los mismos factores traumáticos que en la guerra convencional y también pueden estar sujetos a riesgos físicos y estresores psicológicos asociados con el Trastorno de Estrés Postraumático (TEPT). Según la Teoría de Conservación de los Recursos (COR, por sus siglas en inglés), el TEPT se deriva del agotamiento de los recursos y de la restauración inadecuada.

**Objetivos:** Discutir y metaanalizar los factores relacionados con el TEPT entre el personal de mantenimiento de la paz, basados en la COR, encuadrándolos como recursos o pérdida/amenaza de pérdida de recursos.

**Métodos:** Se realizó una búsqueda sistemática de literatura con palabras clave relevantes, se revisaron 51 artículos y 21 de ellos se metaanalizaron.

**Resultados:** Los factores mencionados en revisiones anteriores, reforzados por los nuestros, incluyen: apoyo familiar/comunitario y militar como recursos; estado civil soltero, género femenino, servicio en la infantería y mayor tiempo desde el despliegue como falta de recursos. Los factores mencionados en revisiones anteriores, confirmados por nuestro metaanálisis, incluyen: educación, rango y afrontamiento centrado en problemas como recursos; percepciones negativas sobre el despliegue, exposición al combate/trauma, factores estresantes del despliegue y duración del despliegue como falta de recursos. Los factores pasados por alto en revisiones anteriores incluyen: la edad como recurso; los eventos negativos de la vida y las interacciones sociales negativas se identificaron como falta de recursos. Las comorbilidades incluyen: problemas de salud física, el impacto posterior al despliegue en el funcionamiento y la psicopatología posterior al despliegue (ej., depresión, uso de sustancias).

**Conclusiones:** Se identificaron significativamente más factores individuales que contextuales. Si bien algunos factores inherentes a las misiones (ej., exposición al combate, factores estresantes del despliegue) no se pueden mitigar, otros son cruciales para prevenir el TEPT del personal de mantenimiento de la paz (p. ej., las estrategias de afrontamiento, la duración del despliegue, las percepciones sobre el despliegue, las interacciones sociales, el apoyo durante el despliegue) y para informar la selección y la monitorización por parte de las Fuerzas Armadas (ej., la psicopatología previa, durante y posterior al despliegue). Sin embargo, los hallazgos deben ser interpretados con cautela debido a las limitaciones (ej. el sesgo de publicación, la heterogeneidad del estudio) que pueden haber afectado la generalización y la fuerza de las recomendaciones.

## 1. Introduction

Post-traumatic stress disorder (PTSD) is a psychopathological condition necessarily linked to a triggering event (Nash et al., 2014), and features in the trauma – related disorders and stressors group of the Diagnostic and Statistical Manual of Mental Disorders fifth edition (American Psychiatric Association, 2013). Military service is a recognised risk factor for PTSD (Greenberg et al., 2008), and a common occurrence in those exposed to war (Maia et al., 2011). In fact, exposure to combat correlates with higher PTSD rates (Fear et al., 2010; Hoge et al., 2004). For instance, United States soldiers, frequently deployed in active war zones, exhibit a higher prevalence of PTSD compared to Portuguese and British soldiers, who primarily participate in peacekeeping operations, thus being less frequently exposed to direct combat situations (Booth-Kewley et al., 2010; Chemtob et al., 1990; Espinoza, 2010; Hing et al., 2012; Hoge et al., 2004; Iversen et al., 2008).

**Figure 1.**
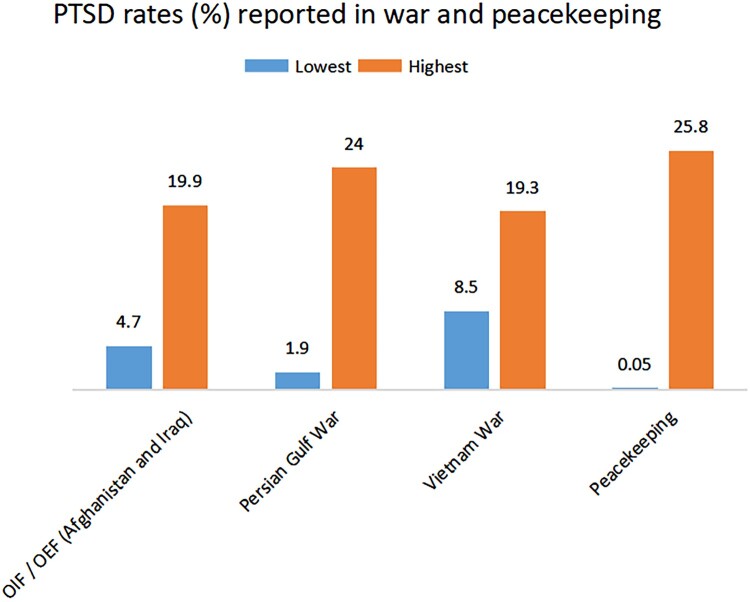
Chart of the PTSD rates (%) reported in war (Source: Magruder & Yeager, 2009) and peacekeeping (Source: Souza et al., 2011).

During peacekeeping operations, soldiers encounter similar traumatic stressors to those of conventional war, e.g., witnessing death and atrocities, and facing hostility and threats from the populations they aim to protect (Greenberg et al., 2008), which may lead to physical risks and psychological stressors associated with PTSD (Greenberg et al., 2008). Furthermore, PTSD rates for peacekeepers match those of soldiers in war (e.g., OIF/OEF, Persian Gulf, Vietnam) (Magruder & Yeager, 2009; Souza et al., 2011) (See Figure 1). However, PTSD rates among peacekeepers vary widely (e.g., Souza et al., 2011) (See Figure 1), which suggests that some factors may either promote or mitigate the development of PTSD.

According to the Conservation of Resources Theory (COR, Hobfoll, 2002), post-traumatic stress disorder arises from the depletion of individual and contextual resources (i.e., objects, personal characteristics and physical, psychological and social conditions that serve to achieve people’s goals), coupled with an inadequate ability to restore those resources, resulting in a state of distress due to the lack of resources required to manage the challenges posed by traumatic experiences. Conversely, resource acquisition and conservation mitigate PTSD by enhancing the individual’s ability to manage the impact of traumatic events (Hobfoll, 2002).

Framed by the COR theory, this systematic review and meta-analysis aim to discuss peacekeepers’ PTSD associated factors, thus offering several contributions. Firstly, while previous reviews (Kaikkonen & Laukkala, 2016; Sareen et al., 2010; Tobin, 2015; Yuan et al., 2024) are atheoretical, merely listing and describing factors correlated with PTSD, this study differs by considering identified PTSD associated factors within the scope of the COR theory, which has the advantage of allowing for an assessment of their role as either resources, protecting peacekeepers from traumatic stress, or loss/threat of loss of resources to cope with traumatic experiences. Secondly, the afore-mentioned reviews examined peacekeepers’ overall health or well-being, whereas this study specifically targets PTSD, which provides a more in-depth, detailed and thorough analysis, improving PTSD research by identifying PTSD specific studies, boosting credibility, and potentially recognising additional PTSD associated factors. Thirdly, while none of the previous reviews conducted a meta-analysis study, this paper does, which has the advantage of providing a quantitative synthesis of data, thereby increasing the statistical power, precision and robustness of the conclusions. Finally, from a practical standpoint, this study will provide guidance to the Armed Forces, regarding the selection, monitoring, and training of peacekeepers, alongside effective mission management strategies, aimed at mitigating and addressing peacekeepers’ PTSD.

## 2. Method

A systematic literature review and meta-analysis were performed to identify PTSD-related factors among peacekeepers. We have preregistered the review in PROSPERO (ID CRD42024581966). Following the Preferred Reporting Items for Systematic Reviews and Meta-Analyses (PRISMA) guidelines (Liberati et al., 2009), the process included four steps: identification, screening, eligibility, and inclusion. The title, abstract, and full text of each article were sequentially analysed. The quality assessment of the reviewed studies was based on the recommendations of the STrengthening the Reporting of OBservational studies in Epidemiology Statement (STROBE; Vandenbroucke et al., 2007), detailed in Appendix A of the Supplemental Material.

**Figure 2.**
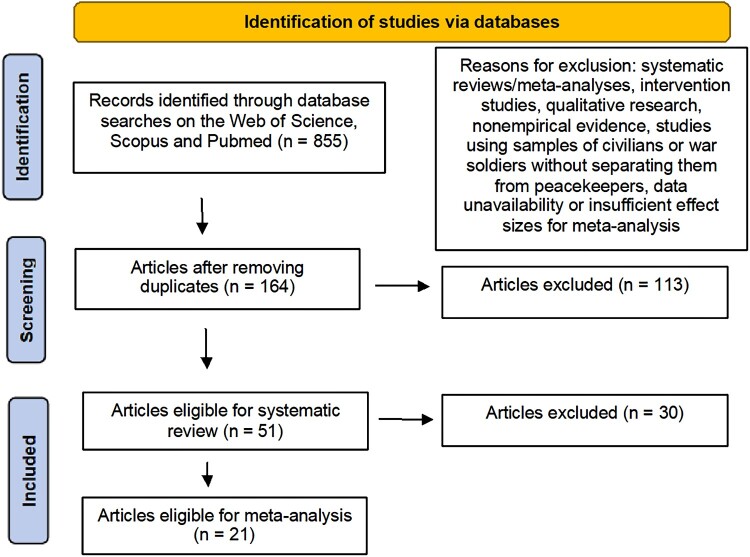
Flow diagram of the systematic selection of studies. Source: adapted from Liberati et al. (2009).

The initial stage of our systematic review involved searching three databases (Web of Science, Scopus, PubMed) using queries with the keywords *PTSD, posttraumatic stress disorder, post-traumatic stress disorder, peacekeepers,* and *peacekeeping*. Articles were required to be written in English and published in relevant research areas (psychology, behavioural science, psychiatry, general internal medicine, medicine). Of the 855 identified articles, 164 were screened after removing duplicates (See Figure 2).

Inclusion and exclusion criteria were set to refine the articles for review (See Figure 2). The inclusion criteria involved studies focusing on military peacekeepers and PTSD, exploring prevalence, explanation, co-occurrence, or consequences of PTSD. The exclusion criteria included systematic reviews/meta-analyses, intervention studies, qualitative research, nonempirical evidence, and studies using samples of civilians or war soldiers without separating them from peacekeepers. Of the 164, 51 articles met these criteria for our systematic review. Of these, 21 were included in the meta-analysis. The remaining 30 were excluded due to data unavailability or insufficient effect sizes for meta-analysis. Requests for data were sent to the authors of 13 articles, however only one response was received, stating data unavailability. The main characteristics of the studies were mapped for both the systematic literature review and the meta-analysis, detailed in Appendices B and C of the Supplemental Material respectively. Some studies identified in previous reviews were not included in our review because these studies did not specifically focus on peacekeepers’ PTSD-related factors.

To code the study's main characteristics, results, and data needed for effect size calculation, a form was prepared, following Lipsey and Wilson's (2001) recommendations. The extracted information included bibliographical details, sample characteristics, study design, variables assessed, main results, and effect sizes. Undisclosed effect sizes in primary studies were calculated using statistical information obtained from the reported data. Appendix D in the Supplemental Material provides a detailed outline of the extracted information.

To assess PTSD-related factors, the Pearson product-moment correlation coefficient (*r*) was calculated for each association. This choice was influenced by the prevalence of correlation studies in the included literature and the measure's interpretability (Rosenthal & DiMatteo, 2001). Additionally, correlations may be derived from various statistical values (e.g., chi-square, *t*, *F*, and *d*), facilitating the transformation of the reported statistics (e.g., odds ratios) (Hunter & Schmidt, 2004). Data were converted into correlation coefficients using the methods and formulas recommended by Lipsey and Wilson (2001) and Borenstein et al. (2009). Multivariate results (e.g., adjusted odds-ratios) were not considered due to their indirect association between variables.

In meta-analytic research, the conversion of correlation coefficients into normally distributed Fisher’s z-values is commonly recommended prior to analysis. All the correlation coefficients were transformed into Fisher’s z-scores prior to analysis and then reconverted into correlations post-analysis for easier interpretation. Effect sizes greater than *r* > .100 were deemed small, *r* > .243 as medium, and *r* > .371 as large, following Rice and Harris’ (2005) guidelines. The direction (positive or negative) of each effect size matched the reported statistical data.

### 2.1. Analysis plan

Two different analytic approaches were used to ensure the assumption of effect sizes’ independence: conventional meta-analysis modelling was performed for each factor with one effect size per study, and three-level multilevel meta-analyses were conducted for factors with multiple effect sizes extracted from the same primary study. These multilevel models allow for modelling three different sources of variance: between studies (level 3), between effect sizes from the same studies (level 2), and between all the retrieved effect sizes (level 1) (Cheung, 2014). All analyses were performed using R Statistical Software (v4.3.1.; R Core Team, 2023). Conventional meta-analyses were performed with the function ‘rma’ of the metafor package (Viechtbauer, 2010), and three-level meta-analytic models were built using the syntax described by Assink and Wibbelink (2016), with the function ‘rma.mv’ of the metafor package (Viechtbauer, 2010).

Moderating variables such as zone of origin and context were included in the models as covariates to explore the variance at levels 2 and 3. Given the potential limitation of having a small number of effect sizes per category of the mediators in each factor, data from individual and contextual factors, and comorbidities were aggregated in two full datasets (risk factors, i.e., correlated with PTSD; and protection factors, i.e., inversely correlated with PTSD) (e.g., Mulder et al., 2018). Before moderation analyses, categories of the discrete variables were transformed into dummy variables.

Finally, nonparametric and funnel-plot based trim-and-fill analyses (Duval, 2005) were performed to diagnose potential biases (such as publication bias). In all analyses, a 5% significant level was used.

## 3. Results

### 3.1. Descriptives

This systematic review analysed 51 articles (See Appendix B). Of those studies, 11 (21.6%) were not identified in the previous reviews (See Appendix E).

Among the studies reviewed, 15 (29.4%) did not specify the average time of PTSD screening in relation to deployment, with evaluation times ranging from 30 days before return to 28 years after. Most studies (45, 88.2%) assessed PTSD post-return, while 2 (3.9%) assessed it pre-return, and 6 (11.8%) evaluated both pre  – and post-return. Various instruments were used to evaluate PTSD, with the post-traumatic stress disorder checklist (PCL, Weathers et al., 1993) being the most common, used in 18 studies (35.2%) alone and in 9 studies (17.6%) alongside another instrument. Sample sizes varied from 50 to 10605 participants, with 25 studies (49%) including over 1,000 participants. PTSD prevalence, analysed in 27 studies (52.9%), ranged from 1.4% to 77.6%. Appendix B provides a detailed outline of the extracted information. It is important to note that this heterogeneity among the included studies introduces significant variability that complicates the interpretation of the overall effect and may limit the generalizability of the findings.

The current meta-analysis examined 22 samples from 21 studies (41.2%, 121 effect sizes; See Appendix C) selected from the systematic review, while the remaining 30 studies (58.8%) were excluded due to data unavailability or insufficient effect sizes for meta-analysis.

### 3.2. Individual factors on post-traumatic stress disorder

A total of 36 studies (70.5%) in the systematic literature review and 18 of those studies (35.3%) in the meta-analysis specifically investigate individual factors (See Appendices B and C).

In the systematic literature review, demographic factors were examined across 11 studies (21.6%). Although 15 studies (29.4%) exclusively featured male samples, and only 12 studies (23.5%) investigated samples with over 10% female representation, findings from 3 studies (5.9%) indicated that being female is associated with higher PTSD rates. Regarding marital status, 7 studies (13.7%) suggested that being single is linked to higher PTSD rates, whereas being married is associated with lower rates. Regarding age, with mean participant ages ranging from 20.9 years to 55 years, 8 studies (15.7%) indicated that older age is associated with lower PTSD rates. Additionally, 5 studies (9.8%) suggested that higher education levels are linked to lower PTSD rates. In the meta-analysis (See Table 1), 4 education studies (19%) and 3 post-deployment age studies (14.3%) showed small negative significant effects (*r* = −.110, *p* = .006 and *r* = −.080, *p* = .027, respectively), confirming that higher education and older age are significantly associated with lower PTSD. However, contrary to the systematic review's findings, the meta-analysis found no significant effects for pre/during-deployment age (3 studies, 14.2%, *p* = .095, *r* = −.090), gender (female) (2 studies, 9.5%, *r* = .034, *p* = .211), and marital status (single) (3 studies, 14.2%, *r* = .067, *p* = .077) on PTSD.

**Table 1.** Results for the overall mean effect sizes of individual factors.

Factor	# Studies	# ES	Fisher’s *z* (SE)	95% CI	Sig. mean *z* (*p*)	Mean *r*	% Var. level 1	Level 2 variance	% Var. level 2	Level 3 variance	% Var. level 3
Age (being older)	3	3	−.080 (.036)	−0.150, −0.009	.027	−.080	–	–	–	–	–
Pre/during-deployment age (being older)	3	3	−.090 (.054)	−0.195, 0.016	.095	−.090	–	–	–	–	–
Gender (being female)	2	2	.034 (.027)	−0.019, 0.088	.211	.034	–	–	–	–	–
Rank	4	4	−.117 (.029)	−0.173, −0.061	< .001	−.116	–	–	–	–	–
Education	4	4	−.110 (.040)	−0.190, −0.032	.006	−.110	–	–	–	–	–
Marital status (being single)	3	3	.067 (.038)	−0.007, 0.142	.077	.067	−	−	−	−	−
Coping strategies	2^1^	10	−.174 (.059)	−0.307, −0.040	.016	−.172	6.94	.032***	93.06	.000	0.00
Previous deployment experience	2	2	.062 (.123)	−0.180, 0.303	.617	.062	–	–	–	–	–
Pre/during-deployment psychopathology	3	4	.225 (.084)	−0.042, 0.493	.075	.221	6.68	.025***	93.32	.000	0.00
Negative life events	4^1^	4	.269 (.055)	0.095, 0.442	.016	.263	–	–	–	–	–
Negative perceptions about deployment	4	8	.212 (.045)	0.107, 0.318	.002	.209	5.79	.007***	62.98	.003	31.23

Note: Variables without ‘pre/during-deployment’ indication refer to variables assessed post-deployment; # Studies = number of studies; # ES = number of effect sizes; SE = standard error; CI = confidence interval for Fisher’s *z*; Sig. mean *z *= level of significance of mean effect size; Mean *r *= mean effect size (Pearson’s correlation); % var = percentage of variance; Level 2 variance = variance between effect sizes within studies; Level 3 variance = variance between studies. ^1^ One of the studies has two samples, and these two samples are regarded as independent studies.

*p < .05; **p < .01; ***p < .001.

In the systematic review, negative life events were examined across 7 studies (13.7%). Findings indicated that adverse traumatic experiences in childhood (1 study, 2%), exposure to trauma throughout life (4 studies, 7.8%), and stressful life events (2 studies, 3.9%) are associated with higher PTSD rates. The meta-analysis included 4 samples from 3 studies (14.2%) on negative life events, with exposure to trauma throughout life (2 studies, 9.5%) and stressful life events (2 samples from 1 study, 4.8%). It revealed a significant medium positive effect (*r* = .263, *p* = .016), indicating that a higher number of negative life events corresponds to higher PTSD rates.

In the systematic review, pre/during-deployment psychopathology was examined across 5 studies (9.8%). Findings suggested that pre-deployment depression (1 study, 2%), pre-deployment PTSD symptoms (2 studies, 3.9%), pre or during-deployment alcohol consumption (2 studies, 3.9%), history of consulting a psychiatrist before joining the army (1 study, 2%), and use of professional help during deployment (1 study, 2%) are associated with higher PTSD rates. However, the meta-analysis of 3 studies on pre/during-deployment psychopathology (14.2%)  – pre-deployment depression (1 study, 4.8%), pre-deployment alcohol (1 study, 4.8%), pre-deployment PTSD symptoms (1 study, 4.8%), and alcohol during service (1 study, 4.8%)  – showed a small and nonsignificant effect (*r* = .062, *p* = .075).

In the systematic review, professional factors were explored across 13 studies (25.5%). Findings suggested that having an infantry function, longer time since deployment and being in reserve/no longer serving or out of the workforce are associated with higher PTSD rates, whereas having military education and a higher rank are associated with lower PTSD rates. However, previous deployment experience (i.e., being deployed on a greater number of missions) showed mixed results, with 4 studies indicating it is associated with higher PTSD rates and 1 study suggesting lower rates. In the meta-analysis, rank was examined across 4 studies (19%), revealing a significant small negative effect (*r* = −.116, *p* < .001), indicating that higher rank is significantly associated with lower PTSD. However, previous deployment experience, analysed in 2 studies (9.5%), showed a small and nonsignificant effect (*r* = .062, *p* = .617).

In the systematic review, negative perceptions about deployment were examined across 8 studies (15.7%). Findings indicated that a lack of mission meaning, negative perception of the mission, frustration with the mission, and perceiving deployment as threatening are associated with higher PTSD rates. In the meta-analysis, negative perceptions about deployment (lack of meaning of the mission  – 1 study, 4.8%; negative perception of the mission  – 4 studies, 19%; perceiving deployment as threatening  – 1 study, 4.8%) were analysed in 4 studies (19%), where a small positive effect (*r* = .209, *p* = .002) was observed. This supports the notion that higher negative perceptions about deployment correspond to higher PTSD rates.

Only one study (19.6%) in the systematic review analysed *coping strategies* and the results suggest that both wishful thinking and accepting responsibility are associated with higher PTSD rates, while seeking social support and organised problem solving are associated with lower rates. The two samples of this study (4.8%) in the meta-analysis confirm this observation, presenting a small negative significant effect (*r* = −.172, *p* = .016).

### 3.3. Contextual factors in post-traumatic stress disorder

A total of 38 studies (74.5%) in the systematic literature review and 15 of those studies (71.4%) in the meta-analysis specifically investigate contextual factors (See Appendices B and C).

In the systematic review, deployment stressors (15 studies, 29.4%) are prominent. Findings show that peacekeeping stressors (e.g., operational environment, non-traumatic stressors, daily mission problems) (13 studies, 25.5%) and time on deployment (2 studies, 3.9%) correlate with PTSD. The meta-analysis (See Table 2) shows small positive effects for peacekeeping stressors (*r* = .220, *p* < .001; 8 samples in 7 studies, 33.3%) and time on deployment (*r* = .140, *p* = .017; 2 studies, 9.5%), indicating that higher stressors and longer deployment are linked to increased PTSD.

**Table 2.** Results for the overall mean effect sizes of contextual factors.

Factor	# Studies	# ES	Fisher’s *z* (SE)	95% CI	Sig. mean *z* (*p*)	Mean *r*	% Var. level 1	Level 2 variance	% Var. level 2	Level 3 variance	% Var. level 3
Time on deployment	2	2	.141 (.059)	0.025, 0.256	.017	.140	–	–	–	–	–
Social support	3	4	−.105 (.058)	−0.290, 0.080	.168	−.105	9.26	.000	0.00	.009	90.74
Exposure to trauma/combat/ warzone	6	9	.183 (.065)	0.034, 0.332	.022	.181	4.48	.008***	28.31	.018	67.21
Peacekeeping stressors	8^1^	15	.224 (.051)	0.115, 0.334	<.001	.220	2.89	.012***	49.28	.012	47.83
Negative social interactions	2^1^	4	.430 (.089)	0.147, 0.712	.017	.405	8.58	.029***	91.42	.000	0.00

Note: Variables without ‘pre/during-deployment’ or ‘on deployment’ indication refer to variables assessed post-deployment; # Studies = number of studies; # ES = number of effect sizes; SE = standard error; CI = confidence interval for Fisher’s *z*; Sig. mean *z *= level of significance of mean effect size; Mean *r *= mean effect size (Pearson’s correlation); % var = percentage of variance; Level 2 variance = variance between effect sizes within studies; Level 3 variance = variance between studies. ^1^ One of the studies has two samples, and these two samples are regarded as independent studies.

*p < .05; **p < .01; ***p < .001.

In the systematic literature review, potentially traumatic events during deployment (20 studies, 39.2%) are prominent. Exposure to trauma/combat/warzone (14 studies, 27.5%) and other traumatic events (e.g., natural disasters, physical/sexual aggression, personal or others’ injury/illness, being attacked, witnessing atrocities) (6 studies, 11.8%) correlate with PTSD. The meta-analysis of exposure to trauma/combat/warzone (6 studies, 28.6%) shows a small positive effect (*r* = .181, *p* = .022), indicating that higher exposure is related to increased PTSD.

In the systematic literature review, social support from family/community (3 studies, 5.9%) correlates with lower levels of PTSD, while separation/isolation from family/friends/country correlates with higher levels. Military social support (e.g., unit perceived quality of leadership, morale, perceived organisational support, self-disclosure and positive reactions from military personnel) (4 studies, 7.8%) also correlates with less PTSD. Negative social interactions (1 study, 2%) correlates with more PTSD. The meta-analysis of social support (3 studies, 14.3%) shows a small, insignificant effect (*r* = −.105, *p* = .168; perceived organisational support  – 1 study, 4.8%; morale  – 1 study, 4.8%; family and community homecoming  – 2 studies, 9.5%), while negative social interactions (2 samples from 1 study, 4.8%) have a significant medium positive effect (*r* = .405, *p* = .017), indicating that higher levels increase PTSD.

### 3.4. Comorbidities associated with post-traumatic stress disorder

A total of 21 studies (41.2%) in the systematic literature review and 14 of those studies (66.7%) in the meta-analysis specifically investigate comorbidities (See Appendices B and C). In the systematic literature review, post-deployment psychopathology is prominent. Findings suggest that depression (10 studies, 19.6%), other psychopathologies (e.g., anxiety, secondary traumatic stress, burnout, suicidal ideation) (3 studies, 5.9%) and alcohol/substance use (5 studies, 9.8%) correlate with more PTSD. The meta-analysis (9 studies, 42.9%) confirms a small significant positive effect (*r* = .048, *p* < .001; depression  – 5 studies, 23.8%; alcohol  – 3 studies, 14.3%; burnout  – 1 study, 4.8%; secondary traumatic stress  – 1 study, 4.8%; suicidal ideation  – 1 study, 4.8%) between PTSD and post-deployment psychopathology.

In the systematic literature review, the impact of post-deployment on functioning (e.g., mental and functional impairment, hostility, anger, insomnia, problems in social functioning, deficit in figurative and logical memory) (11 studies, 21.6%) correlates with PTSD. The meta-analysis (9 studies, 42.9%) shows a medium significant positive effect (*r* = .262, *p* < .001), confirming the association between higher PTSD and greater impact on functioning.

In the systematic literature review, post-deployment physical health problems (e.g., gastrointestinal disorders, musculoskeletal problems, headaches) (7 studies, 13.7%) correlate with higher levels of PTSD. However, the meta-analysis (3 studies, 14.3%) shows a small, insignificant effect (*r* = .070, *p* = .654), refuting the assumption of a link between higher PTSD and physical health problems.

### 3.5. Heterogeneity and moderating effects

Participants’ origins vary across the studies, including the United States (14 studies, 27.5%), Canada (12 studies, 23.5%), Australia (6 studies, 11.8%), Brazil (2 studies, 3.9%), and European countries including the Netherlands (7 studies, 13.7%), the United Kingdom (2 studies, 3.9%), Norway (4 studies, 7.8%), and Italy (3 studies, 5.9%). Deployment locations also vary: 13 studies (25.5%) do not report deployment locations, 12 (23.5%) assess peacekeepers in war contexts, 8 (15.7%) in peace contexts, and 18 (35.3%) in mixed contexts. Three studies assess PTSD prevalence across specific locations: various missions (2%); Cambodia (3.7%), Lebanon (6.2%), former Yugoslavia (8%); Bosnia (4.4%); Bougainville (5.9%); and East Timor (7.2%).

Likelihood-ratio tests show significant variance within studies (level 2) for Zone of Origin and Context in Risk and Protection Factors, and between studies (level 3) for Zone of Origin and Context in Risk Factors (See Table 3). Moderation analyses indicate no significant effects (See Table 4). Thus, Zone of Origin and Context do not appear to affect PTSD levels, implying no significant differences regardless of participants’ origin (North America, Europe, or Others) or context (Peace, War, or Mixed). The considerable heterogeneity across studies underscores the need for caution in interpreting these findings, as they may not be generalisable.

**Table 3.** Results for the overall mean effect sizes of comorbidities.

Factor	# Studies	# ES	Fisher’s *z* (SE)	95% CI	Sig. mean *z* (*p*)	Mean *r*	% Var. level 1	Level 2 variance	% Var. level 2	Level 3 variance	% Var. level 3
Psychopathology	9	16	.520 (.087)	−0.336, 0.705	<.001	.048	0.79	.117***	99.21	.000	0.00
Impact on functioning	9	19	.268 (.060)	0.141, 0.395	<.001	.262	8.99	.004*	11.22	.026*	79.79
Physical health problems	3	5	.070 (.145)	−0.332, 0.473	.654	.070	1.45	.097***	98.55	.000	0.00

Note: Variables without ‘pre/during-deployment’ indication refer to variables assessed post-deployment; # Studies = number of studies; # ES = number of effect sizes; SE = standard error; CI = confidence interval for Fisher’s *z*; Sig. mean *z *= level of significance of mean effect size; Mean *r *= mean effect size (Pearson’s correlation); % var = percentage of variance; Level 2 variance = variance between effect sizes within studies; Level 3 variance = variance between studies.

*p < .05; **p < .01; ***p < .001.

**Table 4.** Results for categorical moderators (bivariate models).

Moderators	# Studies	# ES	Intercept (95% CI) / mean *z* (95% CI)	Mean *r*	*β* (95% CI)	*F* (df1, df2)^a^	*p*^b^	Level 2 variance	Level 3 variance
Risk Factors									
Zone of Origin	23	93				0.329 (2, 90)	.772	.042***	.017**
North America (RC)	11	56	.279 (0.181, 0.377)	.272					
Europe	7	31	.258 (0.136, 0.381)	.252	−0.021 (−0.178, 0.136)				
Others	3	6	.175 (−0.063, 0.412)	.173	−0.104 (−0.361, 0.152)				
Context	19	90				1.857 (2, 85)	.162	.040***	.012*
Peace (RC)	8	39	.331 (0.226, 0.435)	.319					
War	8	38	.188 (0.084, 0.292)	.186	−0.142 (−0.290, 0.005)				
Mixed	3	11	.239 (0.048, 0.431)	.235	−0.091 (−0.310, 0.127)				
Protection Factors									
Zone of Origin	11	28				0.858 (1, 26)	.363	.013***	.000
North America (RC)	6	12	−0.106 (−0.177, – 0.035)	−.106					
Europe	5	16	−0.149 (−0.212, – 0.085)	−.148	−0.043 (−0.138, 0.052)				
Context	11	28				0.328 (2, 25)	.723	.014***	.000
Peace (RC)	7	17	−0.118 (−0.182, – 0.055)	−.117					
War	3	8	−0.170 (−0.249, – 0.070)	−.168	−0.041 (−0.151, 0.069)				
Mixed	1	3	−0.114 (−0.254, 0.026)	−.114	0.004 (−0.149, 0.158)				

Note: # Studies = number of studies; # ES = number of effect sizes; Mean *r* = mean effect size (*r*); CI = conﬁdence interval; *β* = estimated regression coefficient; RC = reference category; Level 2 variance = variance between effect sizes within studies; Level 3 variance = variance between studies.

^+^ *p *< .10; *p < .05; ***p < .001.

^a^ Omnibus test of all regression coefficients in the model.

^b^ *p*-value of the omnibus test.

### 3.6. Trim-and-fill analyses

The trim-and-fill analyses detected bias in three individual factors and four contextual factors due to asymmetrical funnel plot distributions. Overall effects were adjusted by imputing ‘missing’ effect sizes and re-estimating (See Tables 5–7). For individual factors, a higher effect was observed for rank, and lower effects were seen for previous deployment experience, negative perceptions about deployment, and negative life events. For contextual factors, lower effects were observed for time on deployment and combat/trauma exposure. As for comorbidities, a lower effect was observed for physical health problems.

**Table 5.** Results for the overall mean effect sizes of the individual factors after conducting trim-and-fill analyses.

Factor	# Studies	# ES	Fisher’s *z* (SE)	95% CI	Sig. mean z (*p*)	Mean *r*
Age (being older)	–	–	–	–	–	–
Pre/during-deployment age (being older)	–	–	–	–	–	–
Gender (being female)	–	–	–	–	–	–
Rank	5	5	−.139 (.030)	−0.197, −0.081	−.001	−.138
Education	−	−	−	−	−	−
Marital status (being single)	−	−	−	−	−	−
Pre/during-deployment psychopathology	−	−	−	−	−	−
Coping strategies	−	−	−	−	−	−
Previous deployment experience	3	3	−.060 (.142)	−0.338, 0.218	.671	−.060
Negative perceptions about deployment	5	9	.181 (.037)	0.110, 0.253	−.001	.179
Negative life events	5	5	.242 (.050)	0.144, 0.340	<.001	.237

Note: # Studies = number of studies; # ES = number of effect sizes; SE = standard error; CI = confidence interval for Fisher’s *z*; Sig. mean *z *= level of significance of mean effect size; Mean *r *= mean effect size (Pearson’s correlation).

*p < .05; **p < .01; ***p < .001.

**Table 6.** Results for the overall mean effect sizes of the contextual factors after conducting trim-and-fill analyses.

Factor	# Studies	# ES	Fisher’s *z* (SE)	95% CI	Sig. mean z (*p*)	Mean *r*
Time on deployment	3	3	.090 (.065)	−0.037, 0.217	.164	.090
Social support	–	–	–	–	–	–
Exposure to trauma/combat/warzone	8	11	.124 (.054)	0.019, 0.229	.021	.123
Peacekeeping stressors	–	–	–	–	–	–
Negative social interactions	–	–	–	–	–	–

Note: # Studies = number of studies; # ES = number of effect sizes; SE = standard error; CI = confidence interval for Fisher’s *z*; Sig. mean *z *= level of significance of mean effect size; Mean *r *= mean effect size (Pearson’s correlation).

*p < .05; **p < .01; ***p < .001.

**Table 7.** Results for the overall mean effect sizes of the comorbidities after conducting trim-and-fill analyses.

Factor	# Studies	# ES	Fisher’s *z* (SE)	95% CI	Sig. mean z (*p*)	Mean *r*
Post-deployment psychopathology	–	–	–	–	–	–
Impact on functioning	–	–	–	–	–	–
Physical health problems	4	6	−.005 (.138)	−0.275, 0.266	.973	−.005

Note: # Studies = number of studies; # ES = number of effect sizes; SE = standard error; CI = confidence interval for Fisher’s *z*; Sig. mean *z *= level of significance of mean effect size; Mean *r *= mean effect size (Pearson’s correlation).

*p < .05; **p < .01; ***p < .001.

## 4. Discussion

This study systematically reviewed and meta-analysed peacekeepers’ PTSD associated factors in light of the COR theory (Hobfoll, 2002). In line with previous reviews on peacekeepers’ mental ill-being (e.g., Kaikkonen & Laukkala, 2016; Sareen et al., 2010; Tobin, 2015), we found a similar number of studies on individual and contextual factors (36 and 38, respectively). However, significantly more individual factors were identified compared to contextual factors, in keeping with a previous review on adults’ PTSD predictors (Ozer et al., 2003).

### 4.1. Individual factors on post-traumatic stress disorder

Some individual factors were found which, while being valuable for peacekeepers to face traumatic situations, may be considered resources, according to the COR theory (Hobfoll, 2002), presenting a negative correlation with PTSD. For instance, *age* is a resource that allows for different emotional information processing, with older adults reporting less affect and showing reduced negative attentional bias than younger adults (Konnert & Wong, 2015). While previous reviews did not consider age, our review and meta-analysis observed that older peacekeepers have a lower PTSD risk (Bolton et al., 2002; Litz et al., 1997a). Younger age is linked not only to a higher likelihood but also to greater severity of PTSD (Richardson et al., 2007).

*Educational level* and *rank* are also resources as higher-educated and higher-ranking soldiers tend to be more experienced and have stronger group cohesion, thus fostering greater military resilience (Ha & Jue, 2022) and potentially reducing distress (Sareen et al., 2010). Therefore, our review and meta-analysis observed that education level and rank negatively correlate with peacekeepers’ PTSD (Bolton et al., 2001, 2002; Bramsen et al., 2000; Gjerstad et al., 2020; Litz et al., 1997a; Richardson et al., 2007). These factors had already been mentioned in previous reviews (Kaikkonen & Laukkala, 2016; Tobin, 2015) but they are reinforced in our review since Tobin’s review relied solely on one study (Dirkzwager et al., 2005), and Kaikkonen and Laukkala’s review on another (Jones et al., 2013), while ours includes that mentioned by Tobin and six additional studies, and our meta-analysis using four studies confirms these results. Our review also observed that military education in particular, not mentioned in previous reviews, is negatively linked to PTSD (Gjerstad et al., 2020; Greenberg et al., 2008).

*Problem-focused coping strategies* are also resources since, instead of dealing solely with the emotions evoked by the stressor, they deal directly with the stressor, which may facilitate adjustment (Dirkzwager et al., 2003). Thus, our review and meta-analysis observed that problem-focused coping strategies (e.g., seeking social support, organised problem solving) negatively correlate with PTSD, while emotion-focused coping strategies (e.g., wishful thinking, accepting responsibility) positively correlate with PTSD (Dirkzwager et al., 2003). This factor had already been mentioned in a previous review (Sareen et al., 2010), but while it relied on two studies (Dirkzwager et al., 2003; Ippolito et al., 2005), ours, despite using only the former, confirms and provides statistical power to the finding, via a meta-analysis.

Conversely, some individual factors were found which, while threatening the acquisition and conservation of resources, cause resource depletion, and thus, may be considered losses or threats of losses of resources, according to the COR theory (Hobfoll, 2002), presenting a positive correlation with PTSD. For instance, *female gender* facilitate loss or threat of loss of resources situations given the fact that women potentially have higher vulnerability or increased exposure to stressors (Roxburgh, 1996) which may lead to an over-investment (possibly resulting in depletion) of resources to cope. Thus, our review observed that female gender positively correlates with peacekeepers’ PTSD (Adler et al., 2005; Sareen et al., 2008; Yarvis & Schiess, 2008). Despite limited female representation in these studies, the findings indicate that female peacekeepers are 1.977 times more prone to subclinical PTSD than males (Yarvis & Schiess, 2008). This factor had already been mentioned in a previous review (Tobin, 2015) but is reinforced by our review since the previous review did not specify the number of studies, while ours used three studies. Similarly, a *single marital status* should also facilitate a loss or threat of loss of resources as single individuals report lower perceived social support compared to their married counterparts (Soulsby & Bennett, 2015) and may have to over-invest resources to cope with stressors, possibly leading to a depletion of resources. Thus, our review observed that a single marital status positively correlates with peacekeepers’ PTSD (Bolton et al., 2001; Forbes et al., 2016; Greenberg et al., 2008; Richardson et al., 2007; Yarvis & Schiess, 2008). Our findings suggest single peacekeepers are 1.893 times more prone to subclinical PTSD than married peacekeepers (Yarvis & Schiess, 2008), with a single status linked to a higher PTSD likelihood and severity (Richardson et al., 2007), while married peacekeepers have a lower PTSD risk (Bolton et al., 2002; Greenberg et al., 2008). This factor had already been mentioned in a previous review (Tobin, 2015), but is reinforced by our review, since the previous review relied solely on one study (Greenberg et al., 2008), while ours added four more studies. However, marital status and gender effects are not supported in the meta-analysis, possibly due to the limited statistical power from the studies reporting univariate data. This highlights the need for further research for a more in-depth understanding.

*Pre/during-deployment psychopathology* is also a loss or threat of loss of resources as it may make individuals more vulnerable (Schilbach et al., 2023), possibly leaving them depleted of resources to cope. Thus, our review observed that pre/during-deployment psychopathology is positively correlated with peacekeepers’ PTSD (Dirkzwager et al., 2005; Maguen et al., 2004; Maguen et al., 2009; Mehlum et al., 2006; Ward, 1997). Alcohol consumption during service is associated with PTSD (Mehlum et al., 2006), as are pre-deployment depression (Maguen et al., 2004), pre-deployment PTSD (Maguen et al., 2009), history of consulting a psychiatrist before joining the army (Ward, 1997), and seeking professional help during deployment (Dirkzwager et al., 2005). These factors had already been mentioned in previous reviews (Sareen et al., 2010; Tobin, 2015) but are reinforced by our review, since Tobin (2015) relied solely on one study (Dirkzwager et al., 2005) and Sareen et al. (2010) on another (Ward, 1997), while our review included three additional studies. However, our meta-analysis did not support *pre/during-deployment psychopathology* results, likely due to the limited statistical power from only three studies reporting univariate data. Future studies, incorporating moderators (e.g., exposure to combat, coping strategies) are needed for further understanding.

Similarly, *negative life events* (e.g., exposure to lifetime trauma, stressful life events) are also losses or threat of losses of resources since they may lead to a loss of available social support (e.g., problems with family/friends, divorce), which may make them more vulnerable (Schilbach et al., 2023), possibly leaving them depleted of resources to cope. In fact, a meta-analysis on PTSD predictors in the general population (Ozer et al., 2003) proposes that weaker social support systems may increase PTSD susceptibility, drawing parallels with weakened immune systems’ susceptibility to additional illnesses like the flu. Thus, our review and meta-analysis observed that negative life events, not addressed in previous reviews, are positively correlated with peacekeepers’ PTSD (Dirkzwager et al., 2003; Forbes et al., 2016; Maguen et al., 2004).

*Negative perceptions about deployment* (e.g., mission lacking meaning, frustration with the mission) are also losses or threat of losses of resources as viewing missions as meaningless may result in a loss of psychological resources (e.g., purpose, motivation), triggering an over-investment of resources (e.g., time, effort, emotional energy) in trying to find or create meaning, possibly leaving individuals depleted of resources to cope. Thus, negative perceptions about deployment are positively correlated with peacekeepers’ PTSD (Bolton et al., 2002; Dirkzwager et al., 2005; Gray et al., 2004; Litz et al., 1997a, 1997b; Maguen et al., 2004; Mehlum & Weisæth, 2002; Orme & Kehoe, 2014b).

*Infantry function* is a loss or a threat of loss of resources as infantry soldiers often engage in close combat (Department of the Army, 1992), which is associated with PTSD (e.g., Kaikkonen & Laukkala, 2016), and this may trigger an over-investment of resources to cope with traumatic stress, possibly resulting in a depletion of these resources. Thus, our review observed that infantry function, not addressed in previous reviews, is associated with increased PTSD risk (Di Nicola et al., 2007). Similarly, *longer time since deployment* is a loss or threat of loss of resources due to the fact that since it may lead to more post-trauma life events that can hinder re-adjustment and potentially facilitate PTSD (Mehlum & Weisæth, 2002), it may trigger an over-investment of resources to cope with traumatic stress, possibly resulting in a depletion of these resources. Thus, our review observed that longer time since deployment, not addressed in previous reviews, is linked with increased PTSD risk (Dirkzwager et al., 2005; Platania et al., 2020). Furthermore, *being in reserve/outside the workforce* is also a loss or threat of loss of resources. Indeed, since there are challenges in civilian reintegration and post-deployment social functioning, linked to increased mental health issues (Harvey et al., 2011), this situation may trigger an over-investment of resources to cope, possibly resulting in a depletion of these resources. Thus, our review observed that being in reserve/outside the workforce is linked with increased PTSD risk (Forbes et al., 2016; Gjerstad et al., 2020; Richardson et al., 2007). Kaikkonen and Laukkala’s (2016) review showed that reserve soldiers face a higher PTSD risk than regular personnel, and our review extends this observation to peacekeepers, indicating that being in reserve increases the likelihood of PTSD by 112% (Richardson et al., 2007). However, these three factors were not analysed in the meta-analysis due to insufficient data.

Finally, we found one additional individual factor that points to contradictory effects on PTSD, namely *previous deployment experience* (Adler et al., 2005; Di Nicola et al., 2007; Dirkzwager et al., 2005; Richardson et al., 2007; Yarvis & Schiess, 2008). Adler et al. (2005) found first deployments correlated positively with PTSD, and Dirkzwager et al. (2005) showed multiple deployments reduced PTSD risk. However, Richardson et al. (2007) found peacekeepers deployed more than once have higher probable PTSD rates, regardless of location, and Yarvis and Schiess (2008) found peacekeepers deployed multiple times have 3.676 times higher PTSD risk than those never deployed. Sareen et al.’s review (2010) had already noted the contradictory effects of previous deployment experience on peacekeepers’ PTSD, but this contradiction is reinforced by our review since Sareen et al.’s review relied on three studies (Adler et al., 2005; Richardson et al., 2006; Richardson et al., 2007), while ours used two of these studies and an additional three. Deployment experience may benefit soldiers since counterinsurgency experience may provide resources, thus preventing PTSD (Dixit et al., 2018). However, peacekeepers may not accumulate benefits over subsequent deployments (Adler et al., 2005), as multiple deployments may increase exposure to combat trauma (Fear et al., 2010), possibly depleting resources. However, our meta-analysis did not solve these contradictory results, likely due to the limited statistical power from only 2 studies reporting univariate data.

### 4.2. Contextual factors in post-traumatic stress disorder

We found a contextual factor which, while being valuable for peacekeepers to face traumatic situations, may be considered a resource, according to the COR theory (Hobfoll, 2002), presenting a negative correlation with PTSD. *Social support* is a resource that may influence coping (e.g., seeking assistance; Pierce et al., 2013), aiding adjustment (Dirkzwager et al., 2003). Thus, our review observed that family/community (Bolton et al., 2002, 2003; Orme & Kehoe, 2014b) and military support (Barnes et al., 2013; Bolton et al., 2003; Maguen et al., 2004; Mehlum & Weisæth, 2002) is negatively correlated with peacekeepers’ PTSD. This factor had already been observed in Sareen et al.’s review (2010) but is reinforced by our review since Sareen et al.’s (2010) relied on two studies (Bolton et al., 2002; Greenberg et al., 2008), while ours included one of these and a further two for family/community support, and an additional four for military support. However, this was not confirmed by our meta-analysis, possibly due to the limited statistical power (3 studies). Further research is needed to fully understand the role of social support in PTSD.

Conversely, some contextual factors were found which, while threatening the acquisition and conservation of resources, cause resource depletion, and thus, may be considered losses or threats of losses of resources, according to the COR theory (Hobfoll, 2002), presenting a positive correlation with PTSD. For instance, *trauma/combat/warzone* is a loss or threat of loss of resources given that PTSD is inherently linked to traumatic event exposure, as per the Diagnostic and Statistical Manual of Mental Disorders (DSM; American Psychiatric Association, 2013) Criterion A, i.e., PTSD emerges in response to a triggering event (Nash et al., 2014), characterised by overwhelming external stressors (Horwitz, 2018). Thus, our review and meta-analysis observed that trauma/combat/warzone positively correlate with peacekeepers’ PTSD (Bolton et al., 2001, 2002, 2006; Bramsen et al., 2000; Connorton et al., 2011; Di Nicola et al., 2007; Dickstein et al., 2010; Dirkzwager et al., 2005; Forbes et al., 2016; Gjerstad et al., 2020; Gray et al., 2004; Klaassens et al., 2008; Litz et al., 1997b; Maguen et al., 2004, 2009; Orme & Kehoe, 2014a; Sareen et al., 2007, 2013; Seedat et al., 2003; Ward, 1997). This factor had already been mentioned in previous reviews (Kaikkonen & Laukkala, 2016; Sareen et al., 2010; Tobin, 2015), but is reinforced by our review since Kaikkonen and Laukkala (2016) used three studies (Fear et al., 2010; Hassija et al., 2012; Maguen et al., 2010), Sareen et al. (2010) used five studies (Bramsen et al., 2000; Dirkzwager et al., 2005; Litz et al., 1997a; Sareen et al., 2007, 2008) and Tobin (2015) does not specify any study, while ours used 20 studies, meta-analysing six of them.

*Non-traumatic peacekeeping stressors* (e.g., operational environment, daily mission problems) are also a loss or threat of loss of resources since, despite not being traumatic, the stressors challenge the peacekeepers’ access to essential resources (e.g., adequate rest, sense of control, leadership support), possibly leading to an over-investment (and depletion) of resources (e.g., time, attention, resilience) to cope with them. Thus, our review and meta-analysis observed that non-traumatic peacekeeping stressors positively correlate with peacekeepers’ PTSD (Álvares et al., 2020; Bolton et al., 2006; Bramsen et al., 2000; Dickstein et al., 2010; Dirkzwager et al., 2003; Litz et al., 1997a; Maguen et al., 2004; Mehlum et al., 2006; Mehlum & Weisæth, 2002; Orme & Kehoe, 2014a; Orme & Kehoe, 2014b; Souza et al., 2008; Waller et al., 2012). This factor had already been mentioned in previous reviews (Sareen et al., 2010; Yuan et al., 2024) but is reinforced by our review and meta-analysis since Sareen et al. (2010) used one study (Litz et al., 1997a) and Yuan et al. (2024) used five studies (Bolton et al., 2006; Elrond et al., 2019; Fernando et al., 2011; Gjerstad et al., 2020; Litz et al., 1997a), while ours included two of these and a further eleven, meta-analysing seven of them. This aligns with Klaassens et al. (2008), stating trauma exposure explains only 9% of PTSD variance, emphasising the importance of non-traumatic stressors in PTSD development.

*Longer time on deployment* is also a loss or threat of loss since it may heighten exposure to combat or non-traumatic stressors. Thus, our review and meta-analysis observed that longer time on deployment positively correlates with peacekeepers’ PTSD (Adler et al., 2005; Platania et al., 2020). This factor had already been mentioned in a previous review (Sareen et al., 2010), but is reinforced by our review and meta-analysis, since Sareen et al. (2010) used one study, while ours included this and one more, meta-analysing both. Future studies on the moderating effect of exposure to combat or stressors are needed for a more in-depth understanding.

Finally, *negative social interactions* are also a loss or threat of loss of (social) resources since these interactions (e.g., family/friends problems, divorce) can negatively influence coping (e.g., seeking assistance; Pierce et al., 2013) and adjustment (Dirkzwager et al., 2003). Thus, our review and meta-analysis observed that negative social interactions, overlooked in previous reviews, positively correlate with peacekeepers’ PTSD (Dirkzwager et al., 2003).

### 4.3. Comorbidities associated with post-traumatic stress disorder

According to the COR theory (Hobfoll, 2002), comorbidities can exert strain on an individual’s resources as they are threats to the acquisition and conservation of resources, possibly causing resource depletion. Thus, individuals facing comorbidities may experience a compounding effect, where the demands of managing multiple health conditions (i.e., PTSD and others) further deplete their resources, making it increasingly challenging to cope. Previous reviews in the general population have noted comorbidity between PTSD and mental health problems (depression: Morris et al., 2012; Panagioti et al., 2012; anxiety: Coventry et al., 2020; personality disorders: Friborg et al., 2013). A large US soldier cohort study found PTSD often co-occurs with depression, adjustment disorders, anxiety, and alcohol issues (Walter et al., 2018).

Interestingly, peacekeeper reviews lacked an analysis of comorbidities of mental health and PTSD, except Yuan et al. (2024), who included one study examining PTSD and suicidal ideation. Our study, including 13 studies (Adler et al., 2005; Asmundson et al., 2002; Elhai et al., 2011; Gjerstad et al., 2020; Maguen et al., 2004, 2009; Platania et al., 2020; Richardson et al., 2007, 2008; Seedat et al., 2003; Stapleton et al., 2006; Thoresen & Mehlum, 2008; Yarvis & Schiess, 2008) and meta-analysing nine of those, found that post-deployment PTSD relates to *post-deployment psychopathology* (e.g., depression, other psychopathologies, substance use). PTSD symptoms are linked to depression (Asmundson et al., 2002), with peacekeepers with PTSD having a 41.667 times higher likelihood of depressive symptoms (Yarvis & Schiess, 2008). Interrelationships between PTSD dysphoria and depression factors exist, possibly sharing variance (Elhai et al., 2011). However, most studies are cross-sectional, hindering causal determination. Two longitudinal studies (Maguen et al., 2004, 2009) suggest the significant impact of PTSD on depression and alcohol consumption. Further longitudinal research is necessary for a deeper understanding.

Tobin’s review (2015) found PTSD prevalence linked to *post-deployment impact on functioning* (e.g., cognitive, and functional impairment, hostility, anger, insomnia), supported by our review and meta-analysis. However, Tobin used two studies (Orsillo et al., 1996; Ward, 1997), while our study used one of these and a further ten (Bramsen et al., 2000; Forbes et al., 2005; Geuze et al., 2009; Gjerstad et al., 2020; Maguen et al., 2004, 2009; Platania et al., 2020; Richardson et al., 2007; Richardson et al., 2008; Seedat et al., 2003), meta-analysing nine. Peacekeepers with PTSD experience significant deterioration compared to those without (Seedat et al., 2003), affecting their quality of life (Richardson et al., 2008) and cognitive skills (e.g., memory, learning performance) (Geuze et al., 2009), thus highlighting the relationship between PTSD and soldiers’ functioning (Richardson et al., 2008).

Previous reviews (Kaikkonen & Laukkala, 2016; Sareen et al., 2010) have observed peacekeepers’ PTSD linked with *post-deployment physical health*, which is supported by ours. However, Kaikkonen and Laukkala (2016) used one study (Maguen et al., 2012) and Sareen et al. (2010) used four studies (Elhai et al., 2007; Poundja et al., 2006; Richardson et al., 2006; Stapleton et al., 2006), while ours, including two of these, resorted to five additional studies (Asmundson et al., 2002, 2003; Richardson et al., 2008, 2009; Yarvis & Schiess, 2008), meta-analysing three. PTSD is linked not only to physical health issues (e.g., Richardson et al., 2006) but also to chronic pain (Asmundson et al., 2003) and increased recourse to medical services (Stapleton et al., 2006). However, the meta-analysis did not confirm these results, likely due to limited data from three studies.

### 4.4. Heterogeneity and moderating effects

This meta-analysis revealed no significant moderating effects of zone of origin or context. Differences in PTSD across zones (North America, Europe, and Other) and contexts (Peace, War, and Mixed) were not statistically significant, suggesting they do not moderate the relationship between risk/protective factors and PTSD. However, the peace context had a notable impact on PTSD as regards risk factors, indicating a potentially stronger influence during peaceful periods, though not statistically significant.

### 4.5. Trim-and-fill analyses

The trim-and-fill analyses suggested missing data for some factors, indicating true effects may differ from estimated ones. While past research has raised concerns about the algorithm's limitations (e.g., Peters et al., 2007), it is still valuable for assessing sensitivity to publication bias (e.g., Fernández-Castilla et al., 2021). In this case, the analyses even reinforced the effects in some factors (previous deployment experience, physical health problems), suggesting an underestimation of their association with PTSD. Larger datasets are needed for a clearer understanding.

### 4.6. Limitations

Though insightful, this study has limitations that underscore the need for cautious interpretation and might affect the generalizability of the findings. It did not include non-published studies (e.g., dissertations, conference papers), potentially omitting relevant data that could influence the overall conclusions. Although we present trim-and-fill analyses, the reliance on published studies may introduce a bias towards significant findings, as studies with null or negative results are less likely to be published (publication bias) (Song et al., 2010). This selective inclusion could have inflated the effect sizes observed in our meta-analyses, skewing the results and impacting the generalizability and the accuracy of effect size estimates. The search terms used were limited in scope, which may have excluded relevant research. Language bias, particularly the exclusion of non-English studies, might have led to an overrepresentation of research from English-speaking countries, further limiting the global applicability of our findings. Future reviews should broaden the search strategies, encompassing unpublished studies and multiple languages.

Despite efforts to obtain missing data from the original studies, many studies (*n* = 30) lacked sufficient data and were excluded from the analysis. This exclusion not only limits the completeness of the analysis but also introduces a risk of bias, as the missing data may differ from the available data. This constraint potentially overlooks relevant data, further limiting the robustness and generalizability of the findings. Future research should prioritise obtaining and incorporating missing data to enhance the robustness of the findings. Our analysis focused on univariate data (Cohen’s d, Pearson’s correlation, or Odds Ratio), possibly reducing statistical power. Small sample sizes in several studies were noted, potentially limiting the reliability of the findings and heightening the impact of publication bias (e.g., Turner et al., 2013). Furthermore, primary studies are limited since they mainly rely on correlational data, which restricts the ability to infer causality and overlooks potential moderating variables that could influence the observed relationships. For instance, differences in the timing of PTSD screening relative to deployment (pre- or post-return) could have maturation or moderating effects on the reported PTSD prevalence, which were not explored in this review due to significant imbalance in study distribution. Future studies should employ longitudinal or experimental designs and account for potential moderators (e.g., diversify the moment of PTSD assessment, to evaluate the possible effect of this variable on PTSD development).

Heterogeneity was also noted across the included studies, attributed to different participant characteristics, PTSD measurement tools, methodological approaches, time periods and geopolitical contexts. Such variability complicates the interpretation of the overall effect size. Future research should consider employing multivariate and subgroup analyses to address these sources of heterogeneity and to explore potential moderating factors. Additionally, conducting an umbrella review to synthesise the findings from multiple systematic reviews on this topic could provide a more comprehensive understanding of the field.

### 4.7. Implications

Despite its limitations, this study illuminates peacekeepers’ PTSD research, exploring individual and contextual factors influencing its onset, framing these factors within the COR theory, assessing their role as either resources protecting peacekeepers from traumatic stress or loss/threat of loss of resources to cope with traumatic experiences. It reinforces previous findings on peacekeepers’ mental health as by focusing on PTSD more studies are examined, highlighting family/community and military support as resources; single marital status, female gender, serving in infantry, and longer time since deployment, as lack of resources, predicting PTSD; and prior deployment experience as a mixed result, both as a resource and a lack of resources. Furthermore, this study has strengthened prior reviews via meta-analysis, confirming education, rank, and problem-focused coping as resources; and negative perceptions about deployment, combat/trauma exposure, deployment stressors, and deployment duration as lack of resources, predicting PTSD. It has also expanded understanding by highlighting age as a resource; and negative life events, and negative social interactions as lack of resources. These predictors have been overlooked in previous reviews. This review has also enhanced previous findings regarding comorbidities by examining more studies, showing that post-deployment PTSD correlates with physical health problems, and by meta-analysis, confirming that post-deployment PTSD correlates with impact on functioning. It has also expanded previous reviews on peacekeepers and the general population, confirming that post-deployment PTSD is associated with post-deployment psychopathology, including depression and substance use.

From a practical standpoint, this study emphasises that, while some contextual losses, inherent to missions (e.g., combat exposure, deployment stressors), cannot be mitigated, other contextual losses (e.g., time on deployment, perceptions about deployment), as well as individual (e.g., coping strategies) and contextual (e.g., social interactions, support during deployment) resources are crucial for preventing peacekeepers’ PTSD. This study also informs the Armed Forces with prevention and intervention strategies for peacekeepers’ PTSD. The selection, preparation and monitoring of missions must evaluate and invest in the development of resources that foster peacekeepers’ resilience and provide an adequate response to traumatic situations.

## Supplementary Material

Supplemental Material.docx

## Data Availability

The data that support the findings of this study are available from the corresponding author, LC, upon reasonable request.
