# Supplementary material for: Post-traumatic stress disorder in peacekeepers: a systematic literature review and meta-analysis
Source: Eur J Psychotraumatol. 2024 Oct 22;15(1):2413735. doi: 10.1080/20008066.2024.2413735 (PMC11497577; doi:10.1080/20008066.2024.2413735)
Supplement: Supplemental Material.docx [file ZEPT_A_2413735_SM2580.docx]

# Appendix A: Quality assessment^1^ of method reporting in the reviewed studies.

| References | Study design ^a^ | Setting ^b^ | Participants ^c^ | Variables ^d^ | Data sources/ measurement ^e^ | Bias ^f^ | Sample size ^g^ | Quantitative variables ^h^ | Statistical methods ^i^ |
| --- | --- | --- | --- | --- | --- | --- | --- | --- | --- |
| Adler et al. (2005) | ● | ●● | ● | ● | ● | ● | ○ | ● | ●●○○○ |
| Álvares et al. (2020) | ● | ●○ | ○ | ● | ● | ● | ○ | ● | ●◊○○○ |
| Asmundson et al. (2002) | ● | ●● | ● | ● | ● | ● | ○ | ● | ●●○○○ |
| Asmundson et al. (2003), Study 2 | ● | ●● | ● | ● | ● | ● | ○ | ● | ●●○○● |
| Barnes et al. (2013) | ● | ●● | ● | ● | ● | ● | ○ | ● | ●◊●○○ |
| Bolton et al. (2001) | ● | ●○ | ● | ● | ● | ○ | ○ | ● | ●●○○○ |
| Bolton et al. (2002) | ● | ●○ | ● | ● | ● | ● | ● | ● | ●●●●○ |
| Bolton et al. (2003) | ● | ●○ | ● | ● | ● | ● | ● | ● | ●●○○○ |
| Bolton et al. (2006) | ● | ●○ | ● | ● | ● | ● | ● | ● | ●◊○●○ |
| Bramsen et al. (2000) | ● | ●● | ● | ● | ● | ● | ○ | ● | ●◊○○○ |
| Connorton et al. (2011) | ● | ●● | ● | ● | ● | ● | ○ | ● | ●●○○○ |
| Di Nicola et al. (2007) | ● | ●● | ● | ● | ● | ● | ○ | ● | ●●○○○ |
| Dickstein et al. (2010) | ● | ●● | ● | ● | ● | ● | ○ | ● | ●●●●○ |
| Dirkzwager et al. (2003) | ● | ●● | ● | ● | ● | ● | ● | ● | ●●○○○ |
| Dirkzwager et al. (2005a) | ● | ●● | ● | ● | ● | ● | ● | ● | ●●○○○ |
| Dirkzwager et al. (2005b) | ● | ●● | ● | ● | ● | ● | ● | ● | ●●○○○ |
| Elhai et al. (2011) | ● | ●● | ● | ● | ● | ● | ● | ● | ●◊●○○ |
| Engdahl et al. (2011) | ● | ●● | ● | ● | ● | ● | ● | ● | ●●●●○ |
| Forbes et al. (2005) | ● | ●○ | ● | ● | ● | ● | ○ | ● | ●●●●○ |
| Forbes et al. (2016) | ● | ●● | ● | ● | ● | ● | ● | ● | ●●○●○ |
| Geuze et al. (2009) | ● | ●○ | ● | ● | ● | ● | ○ | ● | ●○○○○ |
| Gjerstad et al. (2020b) | ● | ●● | ● | ● | ● | ● | ● | ● | ●◊●●○ |
| Gray et al. (2004) | ● | ●○ | ● | ● | ● | ● | ● | ● | ●●○●○ |
| Greenberg et al. (2008) | ● | ●● | ● | ● | ● | ● | ● | ● | ●●○●● |
| Hotopf et al. (2003) | ● | ●● | ● | ● | ● | ● | ● | ● | ●●○●○ |
| Klaassens et al. (2008) | ● | ●○ | ● | ● | ● | ● | ○ | ● | ●●○●○ |
| Litz et al. (1997a) | ● | ●○ | ● | ● | ● | ● | ○ | ● | ●●○○○ |
| Litz et al. (1997b) | ● | ●○ | ● | ● | ● | ● | ● | ● | ●●○○○ |
| Loscalzo et al. (2018) | ● | ●○ | ● | ● | ● | ● | ○ | ● | ●◊○○○ |
| Maguen et al. (2004) | ● | ●○ | ● | ● | ● | ● | ○ | ● | ●◊○●○ |
| Maguen et al. (2009) | ● | ●● | ● | ● | ● | ● | ○ | ● | ●◊○●○ |
| Mehlum et al. (2006) | ● | ●○ | ● | ● | ● | ● | ○ | ● | ●●○○○ |
| Mehlum and Weisaeth (2002) | ● | ●○ | ● | ● | ● | ● | ● | ● | ●●○○○ |
| Orme & Kehoe (2014a) | ● | ●● | ● | ● | ● | ● | ○ | ● | ●●○○○ |
| Orme & Kehoe (2014b) | ● | ●● | ● | ● | ● | ● | ○ | ● | ●●○○○ |
| Platania et al. (2020) | ● | ●○ | ● | ● | ● | ● | ○ | ● | ●●○○○ |
| Rademaker et al. (2009) | ● | ●● | ● | ● | ● | ● | ○ | ● | ●●○○○ |
| Richardson et al. (2007) | ● | ●● | ● | ● | ● | ● | ● | ● | ●●●○○ |
| Richardson et al. (2008) | ● | ●● | ● | ● | ● | ● | ○ | ● | ●◊●◊○ |
| Richardson et al. (2009) | ● | ●● | ● | ● | ● | ● | ● | ● | ●●●○○ |
| Roemer et al. (1998) | ● | ●○ | ● | ● | ● | ● | ● | ● | ●●●●○ |
| Sareen et al. (2007) | ● | ● | ● | ● | ● | ● | ● | ● | ●●○○○ |
| Sareen et al. (2008) | ● | ● | ● | ● | ● | ● | ○ | ● | ●●○○○ |
| Sareen et al. (2013) | ● | ● | ● | ● | ● | ● | ● | ● | ●●○○○ |
| Seedat et al. (2003) | ● | ●○ | ● | ● | ● | ● | ○ | ● | ●◊○○○ |
| Souza et al. (2008) | ● | ●○ | ● | ● | ● | ● | ○ | ● | ●●○○○ |
| Stapleton et al. (2006) | ● | ●● | ● | ● | ● | ● | ○ | ● | ●●○○○ |
| Thoresen and Mehlum (2008) | ● | ●○ | ● | ● | ● | ● | ● | ● | ●●○○○ |
| Waller et al. (2012) | ● | ●● | ● | ● | ● | ● | ● | ● | ●●○●○ |
| Ward (1997) | ● | ●○ | ● | ● | ● | ● | ● | ● | ●●○○○ |
| Yarvis and Schiess (2008) | ● | ●● | ● | ● | ● | ● | ● | ● | ●●○○○ |
| Note: ^1^The criteria used to assess quality of method reporting are taken from the STrengthening the Reporting of OBservational studies in Epidemiology Statement (STROBE; Vandenbroucke et al., 2007).  ● = met the STROBE recommendation; ○ = did not meet the STROBE recommendation; ◊ not a relevant indicator.  ^a^ Presentation of key elements of study design early in the paper.  ^b^ Two indicators: (1) description of information related to locations; (2) description of information related to periods of recruitment.  ^c^ Description of eligibility criteria, sources and methods of participant selection.  ^d^ Clear definition of outcomes, exposures, predictors, potential confounders.  ^e^ Sources of data and details of measurement methods.  ^f^ Description of any efforts to address potential sources of bias.  ^g^ Description of how the sample size was determined.  ^h^ Explanation of how quantitative variables were managed in the analysis.  ^i^ Five indicators: (1) description of all statistical methods; (2) description of any methods used to examine subgroups and interactions; (3) explanation of missing data management; (4) explanation of how loss to follow-up, matching of cases and controls or sampling strategies were addressed; and (5) description of sensitivity analyses. | | | | | | | | | |

# Appendix B: Included Studies and Main Characteristics - Systematic Review of the Literature

| Authors, year | Time for screening | Instrument of screening PTSD | N | Mean age | Women deployed (%) | PTSD prevalence (%) | Factors assessed |
| --- | --- | --- | --- | --- | --- | --- | --- |
| Adler et al. (2005) | 30 days prior to returning | Post-Traumatic stress scale (Bartone et al., 1994; Castro et al., 1999) | 3339 | NA | 36.7% | 1.9% men, 3.0% women | Rank, gender, longer deployments, first deployment (in men), depression |
| Álvares et al. (2020) | Before and after deployment | PCL-C (Weathers, 1991) | 66 | 24.4 | 0% | NA | Stressful events during the mission |
| Asmundson et al. (2002) | NA | PCL-M (Forbes et al., 2001; Weathers et al., 1993) | 1856 | 53.7 deployed, 49.7 nondeployed | 0% | Approx. 11% deployed, 3% nondeployed | Poor health; poorer health through depression |
| Asmundson et al. (2003), Study 2 | NA | PCL-M (Weathers et al., 1993) | 787 | 49.9 | 0% | Approx. 12% PTSD, 8% PTSD subsyndrome | Chronic pain |
| Barnes et al. (2013) | Before deployment, near the end of deployment, 3-4 and 8-9 months after deployment | PCL (Weathers et al., 1993) | 1039 | 25.28 | 2.8% | NA | Perceived organizational support |
| Bolton et al. (2001) | Prior to deployment | USAMRU-E PTSD Scale (Bartone et al., 1994; Castro & Adler, 1999) | 2947 | 26 | 10% | 5.9% | Rank, marital status, education, number of potential traumatic events |
| Bolton et al. (2002) | 1.5 years after deployment | PCL (Blanchard et al., 1996; Weathers et al., 1993); Mississippi Scale (Keane et al., 1988) | 1023 | 26.68 | 10% | 10.9% | Combat exposure, stressors, family and community homecoming, age, education, marital status, rank |
| Bolton et al. (2003) | 1.5 years after deployment | PCL (Blanchard et al., 1996; Weathers et al., 1993); Mississippi Scale (Keane et al., 1988) | 426 | 26.85 | 0% | 9.4% | Self-disclosure to partner, family, friends and/or military personnel; positive reactions to disclosure |
| Bolton et al. (2006) | 15 weeks after deployment and 1.5 years later | PCL (Blanchard et al., 1996; Weathers et al., 1993); Mississippi Scale (Keane et al., 1988) | 522 | 26.80 | 11% | 8.2% time 1, 10.2% time 2 | Exposure to warzone, peacekeeping stressors |
| Bramsen et al. (2000) | 0-6 years after deployment | SRIP (Hovens et al., 1994) | 572 | 20.9 | 0% | 3% full criteria for PTSD, 21% one criterion | Personality traits of negativism, somatization and psychopathology, exposure to traumatic events during deployment |
| Connorton et al. (2011) | NA | CIDI (Kessler & Ustun, 2004) | 2382 | Exposed, 55 years; unexposed, 43.8 years | 0% | NA | Exposure to combat |
| Di Nicola et al. (2007) | NA | 10-item self-evaluation questionnaire (U.N. Department of Peacekeeping Operations, 1995) | 160 | 25.1 | NA | NA | Economic and other reasons for enrollment, task infantry, other peacekeeping deployments, being attacked |
| Dickstein et al. (2010) | Weeks leading up to deployment to 9 months after deployment | PCL (Blanchard et al., 1996; Weathers et al., 1993) | 635 | 25 | 3% | NA | Depression, alcohol use, aggressive behavior, stress reactivity, military rank, previous traumatic events, combat exposure, peacekeeping daily hassles, resilience |
| Dirkzwager et al. (2003) | 1^st^ group: 13-19 years afer deployment; 2^nd^ group: 3-8 years afer deployment | SRIP (Hovens et al., 2000; Hovens et al., 1994) | 1^st^ group, 311; 2^nd^ group, 499 | NA | NA | NA | Wishful thinking, accepting responsibility, stressors during deployment, positive and negative social interactions, life events prior and during deployment, seeking social support, organized problem solving, additional stressful events |
| Dirkzwager et al. (2005a) | 6.1 years after deployment (average) | SRIP (Hovens et al., 2000; Hovens et al., 1994) | 3481 | 31.0 | 2% | 5.6% PTSD, 5.7% partial PTSD, 9.8% 1 criterion. 8.0% former Yugoslavia, 6.2% Lebanon, 3.7% Cambodia, 2% various missions | Education, marital status, feelings of powerlessness, use of professional help in deployment, perceiving deployment as threatening, number of years elapsed since deployment, potential traumatic events during deployment, deployment in various missions |
| Dirkzwager et al. (2005b) | 6.6 years after deployment (average) | SRIP (Hovens et al., 1994) | 708 partners and 332 parents | PK: 32 | 0% | NA | Partners: PTSD, sleeping and somatic problems, worse quality of marital relationship, negative social support reported. Mothers: PTSD, sleeping and somatic problems |
| Elhai et al. (2011) | NA | PCL-M (Weathers et al., 1993) | 740 | 44.92 | 4.3% | NA | Depression |
| Engdahl et al. (2011) | 0-9 years after deployment | PCL-M (Weathers et al., 1993) | 1066 | 45.33 | 8% | NA | Peacekeeping deployment |
| Forbes et al. (2005) | NA | PCL (Weathers et al., 1993); CAPS (Blake et al., 1995) | 66 | 35.68 | NA | NA | Anger |
| Forbes et al. (2016) | NA | CIDI 3.0 (Andrews & Peters, 1998) | 1025 | 46.5 | 4.5% | 16.8% | Income level, employment status, age, marital status, exposure to lifetime potential traumatic events, fear/terror in potential traumatic events of deployment, being out of the workforce |
| Geuze et al. (2009) | 0-25 years after deployment | CAPS (Blake et al., 1995) | 50 | PTSD group: 33.32. Control: 34.80 | 0% | NA | Figural and logical memory, performance on measures of learning and immediate and delayed verbal memory |
| Gjerstad et al. (2020b) | 28 years after deployment (average) | PCL-M (Weathers et al., 1993) | 10605 | NA | 2.9% | 6.2% | Employment status, traumatic exposure, post-deployment stressors, civilian educational attainment, military education, rank, anxiety, depression, insomnia, drug misuse |
| Gray et al. (2004) | 18 months after deployment (average) | PCL (Weathers et al., 1993); Mississippi Scale for combat-related PTSD (Keane et al., 1988) | 1040 | 26.73 | 6% | 4.5% acute-onset PTSD, 2% remitted, 6.5% delayed-onset PTSD | Perceived lack of meaningfulness of the mission, positive and negative perceptions of the mission, warzone exposure |
| Greenberg et al. (2008) | 6 months - 11 years after deployment | PCL-M (Weathers et al., 1993); PTSR (Greenberg et al., 2008) | 1245 | 36 | 16% | 5.4% (cutoff 44), 3.6% (cutoff 50) | Marital status, being an officer |
| Hotopf et al. (2003) | 6-8 years after deployment | PTSR (Hotopf et al., 2003) | 4404 | 29.3 | 10.6% | 4.4% |  |
| Klaassens et al. (2008) | 10-25 years after deployment | BSI (Derogatis & Melisaratos, 1983) | 729 | 43.8 | 1% | NA | Trauma exposure |
| Litz et al. (1997a) | 19.6 weeks after deployment (average) | PCL (Weathers et al., 1993); Mississippi Scale for combat-related PTSD (Keane et al., 1988) | 3461 | 26.0 | 6.8% | 8% | Frustrations with peacekeeping, warzone stress, frequency of warzone stressor events, education, positive military experience, rewards of military service |
| Litz et al. (1997b) | 15 weeks after deployment (average) | Modified version of PCL (original: Weathers et al., 1993); adaptation of Mississippi scale for combat-related PTSD (original: Keane et al., 1988) | 3310 | 26.02 | 7% | NA | Traditional combat, positive and negative aspects of peacekeeping |
| Loscalzo et al. (2018) | NA | Psychological treatment inventory: PTSD scale (Gori et al., 2015) | 227 | PK: 31.7; Control: 30.67 | PK: 3.6%. controls: 46.7% | NA | Deployment |
| Maguen et al. (2004) | 2-3 weeks before deployment; 7 months after deployment (average) | PCL (Weathers et al., 1993) | 203 | 28.30 | 7% | 4% after deployment, 13% during predeployment interval | Potential traumatic events, post-deployment potential traumatic events, stressors, negative aspects of peacekeeping, assessment of peacekeeping, predeployment PTSD symptoms, predeployment and postdeployment depression, predeployment and postdeployment hostility, postdeployment alcohol; morale |
| Maguen et al. (2009) | 2-3 weeks prior to deployment; 7 months after return (average) | PCL (Weathers et al., 1993) | 203 | 28.30 | 7% | NA | Predeployment PTSD symptoms, trauma exposure, functional impairment, postdeployment violent behavior, postdeployment problem drinking; rank |
| Mehlum et al. (2006) | 4.3 years after deployment | PTSS-10 (Raphael et al., 1989) | 187 | 39.7 | 0% | NA | Problems with social adaptation after homecoming, alcohol consumption during and after deployment, warzone stressors, observer mission, stress exposure during deployment |
| Mehlum and Weisaeth (2002) | 6.6 years after | PTSS-10 (Raphael et al., 1989) | 1624 | CR, 34.5; repatriated, 35.7; control, 34.6 | 2.3% CR, 0.6% repatriated, 1.4% controls | 5% overall, 16% prematurely repatriated | Perceived lack of meaningfulness of the mission, alcohol consumption after deployment, service stress, stressful life events, perceived quality of leadership in the unit |
| Orme & Kehoe (2014a) | 1^st^ day of deployment; end of deployment; after deployment | PCL-C (Weathers et al., 2001) | 448 | 29 | 0% | NA | Traumatic exposure, deployment stressors, operational environment, work, isolation, hardiness |
| Orme & Kehoe (2014b) | During and after deployment | PCL-C (Weathers et al., 2001) | 350 | 29.7, 30.4, and 29.1 (3 groups) | 0% | NA | Work, isolation, operation environment, negative deployment |
| Platania et al. (2020) | 0-36 months after deployment | Davidson Trauma Scale (Davidson et al., 1997) | 399 | 30.94 | 34.6% | NA | Duration of the mission, how long ago mission ended, dispositional resilience, secondary traumatic stress, burnout, compassion satisfaction |
| Rademaker et al. (2009) | NA | SRIP (Hovens et al., 2000) | 120 | PTSD group: Lebanon, 40; former Yugoslavia, 32; areas such as Angola, Iraq, or Cambodia, 34; reference group: 35 | NA | NA | Scales of MMPI-2 |
| Richardson et al. (2007) | 0-9 years after deployment | PCL-M (Weathers et al., 1993) | 1016 | 45.86 | 0% | 10.33% probable PTSD, 3.99% never deployed, 10.92% deployed once, 14.84% deployed more than once. | Age, marital status, number of deployments, life stressors, serving status, current stress, depression |
| Richardson et al. (2008) | NA | CAPS (Blake et al., 1990) | 125 | 41.49 | 0% | 77.6% | General health and physical role, vitality, social functioning, emotional role, mental health impairment, depression |
| Richardson et al. (2009) | 0-9 years after deployment | PCL-M (Weathers et al., 1996) | 707 | 44.87 | 4.2% | 60% | Gastrointestinal disorders, musculoskeletal problems and headaches |
| Roemer et al. (1998) | 0-12 months after deployment; 1-3 years after deployment | Mississippi scale for combat-related PTSD (Keane et al., 1988); PCL (Blanchard et al., 1996; Weathers et al., 1993) | 460 | 26.7 | 8% | NA | Reports of warzone exposure |
| Sareen et al. (2007) | NA | CCHS-CFS (Gravel & Beland, 2005) | 8441 | NA | 14.7% | 2.3% | Exposure to combat, witnessing atrocities |
| Sareen et al. (2008) | NA | PTSD based on exposure to a list of possible traumatic events (Sareen et al., 2008) | 8441 | NA | 30.7% | 2.2% men, 3.3% women | Gender, peacekeeping deployment |
| Sareen et al. (2013) | NA | WHO-CIDI 2.1 (Kessler et al., 2004) | 8340 | Between 18 and 54 | 30.7% | NA | Deployment related traumatic experiences, childhood traumatic experiences |
| Seedat et al. (2003) | Immediately after return | CAPS (Blake et al., 2000) | 198 | 29 | 3% | 25.8% | Exposure to life-threatening injury, sudden violent death of someone close, physical assault, lifetime traumatic exposure, transportation accidents, number of inflicted traumas, distress and impairment, depression, excessive alcohol use, cannabis use |
| Souza et al. (2008) | Immediately after return | PCL-M (Weathers et al., 1993) | 138 | 28.7 | 0% | 1.4% | Negative affect, number of intensively stressful situations in deployment |
| Stapleton et al. (2006) | NA | PCL-M (Weathers et al., 1993) | 473 | 52.42 | 6.6% | 30% | Visits to any health care professional; visits to general practitioners, specialist visits, pharmacist visits, visits to mental health professionals, depression |
| Thoresen and Mehlum (2008) | 6.6 years after deployment (average) | PTSS-10 (Raphael et al., 1989) | 1172 | Suicidal ideation, 33.4; non suicidal ideation, 33.9 | 0% | NA | Suicidal ideation |
| Waller et al. (2012) | 8 years after deployment (average) | PCL-C (Weathers et al., 1993) | 3037 | NA | Bougainville, 14%; East Timor, 11.2% | East Timor, 7.2%; Bougainville, 5.% | Subjective stressors, non-traumatic deployment stressors (work frustration, other people, cultural stressors) |
| Ward (1997) | 15 months after deployment | GHQ-28 (Goldberg & Williams, 1988); IES (Horowitz et al., 1979) | 117 | 24.7 | NA | 20% | History of consulting a psychiatrist prior to joining the army, combat exposure |
| Yarvis and Schiess (2008) | NA | PCL-M (Weathers et al., 1993) | 1968 | NA | 5.7% | 30% | Marital status, gender, number of deployments, alcohol misuse and abuse, depressive symptoms, health problems |
| NA: Not available. PCL: Post-traumatic stress disorder checklist (PCL-M: military version; PCL-C: civilian version). PTSS-10: Posttraumatic symptom scale - 10 items. SRIP: self-rating inventory for PTSD. GHQ: General health questionnaire. IES: Impact of events scale. CAPS: clinician administered PTSD scale. CIDI: Composite International Diagnostic Interview. PSTR: Pot-traumatic stress reaction. BSI: Brief Symptom Inventory. | | | | | | | |

# Appendix C: Included Studies and Main Characteristics - Meta-Analysis

| Authors, year | N | Country of origin | Context of deployment | Individual / Contextual | Risk / Protection | Category | Factor assessed | Effect size (*r*) |
| --- | --- | --- | --- | --- | --- | --- | --- | --- |
| Adler et al., 2005 | 3339 | North America (US) | Peace | Individual | Protection | Higher rank | Rank | -0,07 |
| Adler et al., 2005 | 3339 | North America (US) | Peace | Individual | Risk | Post-deployment psychopathology | Depression | 0,655 |
| Adler et al., 2005 | 3339 | North America (US) | Peace | Individual | Risk | Post-deployment psychopathology | Depression cutoff | 0,53 |
| Adler et al., 2005 | 3339 | North America (US) | Peace | Individual | Risk | Previous deployment experience | Deployment experience | -0,06 |
| Adler et al., 2005 | 3339 | North America (US) | Peace | Contextual | Risk | Time on deployment | Months on current deployment | 0,09 |
| Barnes et al., 2013 | 1039 | North America (US) | Peace | Contextual | Protection | Social support | Perceived organizational support time 2 | -0,077 |
| Bolton et al., 2002 | 1023 | North America (US) | War | Individual | Protection | Age | Age | -0,15 |
| Bolton et al., 2002 | 1023 | North America (US) | War | Individual | Risk | Being female | Gender (female) | 0,03 |
| Bolton et al., 2002 | 1023 | North America (US) | War | Individual | Risk | Being single | Marital status: single | 0,08 |
| Bolton et al., 2002 | 1023 | North America (US) | War | Contextual | Risk | Potentially traumatic events during deployment | Combat exposure scale | 0,28 |
| Bolton et al., 2002 | 1023 | North America (US) | War | Individual | Protection | Education | Education | -0,17 |
| Bolton et al., 2002 | 1023 | North America (US) | War | Individual | Protection | Higher rank | Rank | -0,19 |
| Bolton et al., 2002 | 1023 | North America (US) | War | Contextual | Risk | Negative perceptions | Negative peacekeeping | 0,19 |
| Bolton et al., 2002 | 1023 | North America (US) | War | Contextual | Protection | Social support | Family homecoming | -0,21 |
| Bolton et al., 2002 | 1023 | North America (US) | War | Contextual | Protection | Social support | Community homecoming | -0,21 |
| Bolton et al., 2006 | 522 | North America (US) | War | Contextual | Risk | Potentially traumatic events during deployment | Warzone exposure scale Time 1 | 0,27 |
| Bolton et al., 2006 | 522 | North America (US) | War | Contextual | Risk | Potentially traumatic events during deployment | Warzone exposure scale Time 1 | 0,23 |
| Bolton et al., 2006 | 522 | North America (US) | War | Contextual | Risk | Peacekeeping Stressors | Peacekeeping stressors time 1 | 0,25 |
| Bolton et al., 2006 | 522 | North America (US) | War | Contextual | Risk | Peacekeeping Stressors | Peacekeeping stressors time 1 | 0,22 |
| Bramsen et al., 2000 | 572 | Europe (Netherlands) | War | Individual | Protection | Age pre/during mission | Age pre/during mission | -0,17 |
| Bramsen et al., 2000 | 572 | Europe (Netherlands) | War | Individual | Protection | Education | Education | -0,1 |
| Bramsen et al., 2000 | 572 | Europe (Netherlands) | War | Individual | Risk | Impact of functioning | MMPI Negativism | 0,19 |
| Bramsen et al., 2000 | 572 | Europe (Netherlands) | War | Individual | Risk | Impact of functioning | MMPI Somatization | 0,14 |
| Bramsen et al., 2000 | 572 | Europe (Netherlands) | War | Individual | Risk | Impact of functioning | MMPI Shyness | 0,04 |
| Bramsen et al., 2000 | 572 | Europe (Netherlands) | War | Individual | Risk | Impact of functioning | MMPI Psychopathology | 0,21 |
| Bramsen et al., 2000 | 572 | Europe (Netherlands) | War | Individual | Risk | Impact of functioning | MMPI lack of extroversion | 0,07 |
| Bramsen et al., 2000 | 572 | Europe (Netherlands) | War | Contextual | Risk | Peacekeeping Stressors | Number of stressores during deployment | 0,31 |
| Dirkzwager et al., 2003 - sample 1 | 291 | Europe (Netherlands) | Peace | Individual | Protection | Coping strategies | Planful problem solving time 1 | 0 |
| Dirkzwager et al., 2003 - sample 2 | 471 | Europe (Netherlands) | Peace | Individual | Protection | Coping strategies | Planful problem solving time 1 | -0,08 |
| Dirkzwager et al., 2003 - sample 1 | 291 | Europe (Netherlands) | Peace | Individual | Protection | Coping strategies | Seeking social support time 1 | 0,02 |
| Dirkzwager et al., 2003 - sample 2 | 471 | Europe (Netherlands) | Peace | Individual | Protection | Coping strategies | Seeking social support time 1 | -0,07 |
| Dirkzwager et al., 2003 - sample 1 | 291 | Europe (Netherlands) | Peace | Individual | Protection | Coping strategies | Positive reappraisal time 1 | -0,1 |
| Dirkzwager et al., 2003 - sample 2 | 471 | Europe (Netherlands) | Peace | Individual | Protection | Coping strategies | Positive reappraisal time 1 | 0 |
| Dirkzwager et al., 2003 - sample 1 | 291 | Europe (Netherlands) | Peace | Individual | Protection | Coping strategies | Wishful thinking time 1 | -0,43 |
| Dirkzwager et al., 2003 - sample 2 | 471 | Europe (Netherlands) | Peace | Individual | Protection | Coping strategies | Wishful thinking time 1 | -0,42 |
| Dirkzwager et al., 2003 - sample 1 | 291 | Europe (Netherlands) | Peace | Individual | Protection | Coping strategies | Accepting responsibility time 1 | -0,26 |
| Dirkzwager et al., 2003 - sample 2 | 471 | Europe (Netherlands) | Peace | Individual | Protection | Coping strategies | Accepting responsibility time 1 | -0,32 |
| Dirkzwager et al., 2003 - sample 1 | 291 | Europe (Netherlands) | Peace | Contextual | Risk | Negative life events | Life events in year prior to time 1 (1 year post) | 0,31 |
| Dirkzwager et al., 2003 - sample 2 | 471 | Europe (Netherlands) | Peace | Contextual | Risk | Negative life events | Life events in year prior to time 1 (1 year post) | 0,34 |
| Dirkzwager et al., 2003 - sample 1 | 291 | Europe (Netherlands) | Peace | Contextual | Risk | Negative social interactions | Positive social interactions time 1 (lack of) | 0,32 |
| Dirkzwager et al., 2003 - sample 2 | 471 | Europe (Netherlands) | Peace | Contextual | Risk | Negative social interactions | Positive social interactions time 1 (lack of) | 0,23 |
| Dirkzwager et al., 2003 - sample 1 | 291 | Europe (Netherlands) | Peace | Contextual | Risk | Negative social interactions | Negative social interactions time 1 | 0,5 |
| Dirkzwager et al., 2003 - sample 2 | 471 | Europe (Netherlands) | Peace | Contextual | Risk | Negative social interactions | Negative social interactions time 1 | 0,54 |
| Dirkzwager et al., 2003 - sample 1 | 311 | Europe (Netherlands) | Peace | Contextual | Risk | Peacekeeping Stressors | Deployment stressors | 0,33 |
| Dirkzwager et al., 2003 - sample 2 | 311 | Europe (Netherlands) | Peace | Contextual | Risk | Peacekeeping Stressors | Deployment stressors | 0,36 |
| Dirkzwager et al., 2005a | 3481 | Europe (Netherlands) | Mixed | Individual | Protection | Age | Age | -0,07 |
| Dirkzwager et al., 2005a | 3481 | Europe (Netherlands) | Mixed | Individual | Risk | Being single | Marital status: single | 0,12 |
| Dirkzwager et al., 2005a | 3481 | Europe (Netherlands) | Mixed | Individual | Protection | Education | Education | -0,16 |
| Dirkzwager et al., 2005a | 3481 | Europe (Netherlands) | Mixed | Individual | Protection | Higher rank | Rank | -0,11 |
| Dirkzwager et al., 2005a | 3481 | Europe (Netherlands) | Mixed | Contextual | Risk | Negative perceptions | Mission has become meaningless | 0,25 |
| Dirkzwager et al., 2005a | 3481 | Europe (Netherlands) | Mixed | Contextual | Risk | Negative perceptions | Evaluating deployment as threatening | 0,3 |
| Dirkzwager et al., 2005a | 3481 | Europe (Netherlands) | Mixed | Contextual | Risk | Peacekeeping Stressors | Number of stressors during deployment | 0,35 |
| Dirkzwager et al., 2005a | 3481 | Europe (Netherlands) | Mixed | Contextual | Risk | Peacekeeping Stressors | Insufficient possibilities to intervene | 0,27 |
| Dirkzwager et al., 2005a | 3481 | Europe (Netherlands) | Mixed | Contextual | Risk | Peacekeeping Stressors | Having no control over the situation | 0,28 |
| Dirkzwager et al., 2005a | 3481 | Europe (Netherlands) | Mixed | Contextual | Risk | Peacekeeping Stressors | More feelings of powerlessness | 0,37 |
| Elhai et al., 2011 | 740 | North America (Canada) | War | Individual | Risk | Post-deployment psychopathology | (lack of) Positive Affect | 0,04 |
| Elhai et al., 2011 | 740 | North America (Canada) | War | Individual | Risk | Post-deployment psychopathology | Depressive Affect | 0,6575 |
| Elhai et al., 2011 | 740 | North America (Canada) | War | Individual | Risk | Post-deployment psychopathology | Somatic Complaints | 0,705 |
| Elhai et al., 2011 | 740 | North America (Canada) | War | Individual | Risk | Post-deployment psychopathology | Interpersonal Problems | 0,56 |
| Forbes et al., 2005 | 66 | Others (Australia) | Mixed | Individual | Risk | Impact of functioning | Anger | 0,07 |
| Forbes et al., 2016 | 1025 | Others (Australia) | Mixed | Contextual | Risk | Potentially traumatic events during deployment | Potential traumatic events / trauma exposure | 0,13 |
| Forbes et al., 2016 | 1025 | Others (Australia) | Mixed | Contextual | Risk | Potentially traumatic events during deployment | Fear and/or horror from deployment exposures at the time | 0,27 |
| Forbes et al., 2016 | 1025 | Others (Australia) | Mixed | Contextual | Risk | Negative life events | Lifetime potentially traumatic events | 0,29 |
| Gray et al., 2004 | 1040 | North America (US) | War | Contextual | Risk | Potentially traumatic events during deployment | Warzone exposure | 0,02 |
| Gray et al., 2004 | 1040 | North America (US) | War | Contextual | Risk | Potentially traumatic events during deployment | Warzone exposure | -0,15 |
| Gray et al., 2004 | 1040 | North America (US) | War | Contextual | Risk | Negative perceptions | Positive peacekeeping | 0,131 |
| Gray et al., 2004 | 1040 | North America (US) | War | Contextual | Risk | Negative perceptions | Positive peacekeeping | 0,13 |
| Gray et al., 2004 | 1040 | North America (US) | War | Contextual | Risk | Negative perceptions | Negative peacekeeping | 0,26 |
| Gray et al., 2004 | 1040 | North America (US) | War | Contextual | Risk | Negative perceptions | Negative peacekeeping | 0 |
| Gray et al., 2004 | 1040 | North America (US) | War | Contextual | Risk | Peacekeeping Stressors | Pressure to uphold restraint | 0 |
| Gray et al., 2004 | 1040 | North America (US) | War | Contextual | Risk | Peacekeeping Stressors | Pressure to uphold restraint | -0,02 |
| Maguen et al., 2004 | 1132 | North America (US) | Peace | Contextual | Risk | Potentially traumatic events during deployment | Potential traumatic events / trauma exposure | 0,34 |
| Maguen et al., 2004 | 1132 | North America (US) | Peace | Individual | Risk | Impact of functioning | Post-deployment hostility | 0,5 |
| Maguen et al., 2004 | 1132 | North America (US) | Peace | Contextual | Risk | Negative life events | Lifetime potentially traumatic events | 0,12 |
| Maguen et al., 2004 | 1132 | North America (US) | Peace | Contextual | Risk | Negative perceptions | Negative aspects of peacekeeping | 0,28 |
| Maguen et al., 2004 | 1132 | North America (US) | Peace | Contextual | Protection | Social support | Morale | -0,02 |
| Maguen et al., 2004 | 1132 | North America (US) | Peace | Individual | Risk | Post-deployment psychopathology | Post-deployment alcohol | 0,47 |
| Maguen et al., 2004 | 1132 | North America (US) | Peace | Individual | Risk | Post-deployment psychopatholoy | Post-deployment depression | 0,78 |
| Maguen et al., 2004 | 1132 | North America (US) | Peace | Individual | Risk | Pre/during deployment psychopathology | Pre-deployment depression | 0,37 |
| Maguen et al., 2004 | 1132 | North America (US) | Peace | Individual | Risk | Pre/during deployment psychopathology | Pre-deployment alcohol | 0,09 |
| Maguen et al., 2004 | 1132 | North America (US) | Peace | Contextual | Risk | Peacekeeping Stressors | Peacekeeping stressors | 0,47 |
| Maguen et al., 2004 | 1132 | North America (US) | Peace | Contextual | Risk | Peacekeeping Stressors | General oversea military stressors | 0,1 |
| Maguen et al., 2009 | 203 | North America (US) | Peace | Individual | Protection | Age pre/during mission | Age pre/during mission | -0,025 |
| Maguen et al., 2009 | 203 | North America (US) | Peace | Contextual | Risk | Potentially traumatic events during deployment | Trauma exposure | 0,08 |
| Maguen et al., 2009 | 203 | North America (US) | Peace | Individual | Protection | Higher rank | Rank | -0,1 |
| Maguen et al., 2009 | 203 | North America (US) | Peace | Individual | Risk | Impact of functioning | Functional impairment | 0,42 |
| Maguen et al., 2009 | 203 | North America (US) | Peace | Contextual | Risk | Impact of functioning | Violence | 0,375 |
| Maguen et al., 2009 | 203 | North America (US) | Peace | Individual | Risk | Post-deployment psychopathology | Post-deployment alcohol | 0,4375 |
| Maguen et al., 2009 | 203 | North America (US) | Peace | Individual | Risk | Pre/during deployment psychopathology | Previous PTSD symptoms | 0,3325 |
| Mehlum et al., 2006 | 187 | Europe (Norway) | War | Individual | Protection | Age pre/during mission | Age pre/during mission | -0,03 |
| Mehlum et al., 2006 | 187 | Europe (Norway) | War | Individual | Risk | Impact of functioning | Social adaptation problems | 0,17 |
| Mehlum et al., 2006 | 187 | Europe (Norway) | War | Individual | Risk | Pre/during deployment psychopathology | Alcohol during service | 0,07 |
| Mehlum et al., 2006 | 187 | Europe (Norway) | War | Contextual | Risk | Peacekeeping Stressors | Service stress index | 0,11 |
| Platania et al., 2020 | 399 | Europe (Italy) | War | Individual | Risk | Being female | Gender (female) | 0,0466 |
| Platania et al., 2020 | 399 | Europe (Italy) | War | Individual | Risk | Impact of functioning | (lack of) dispositional resilience | 0,32 |
| Platania et al., 2020 | 399 | Europe (Italy) | War | Individual | Risk | Impact of functioning | (lack of) compassion satisfaction | 0,42 |
| Platania et al., 2020 | 399 | Europe (Italy) | War | Individual | Risk | Post-deployment psychopathology | Burnout | 0,25 |
| Platania et al., 2020 | 399 | Europe (Italy) | War | Individual | Risk | Post-deployment psychopathology | Secondary traumatic stress | 0,16 |
| Platania et al., 2020 | 399 | Europe (Italy) | War | Contextual | Risk | Time on deployment | Duration of operation (> 9 months) | 0,2066 |
| Richardson et al., 2007 | 1016 | North America (Canada) | Peace | Individual | Protection | Age | Age | -0,02 |
| Richardson et al., 2007 | 1016 | North America (Canada) | Peace | Individual | Risk | Being single | Marital status | -0,008 |
| Richardson et al., 2007 | 1016 | North America (Canada) | Peace | Individual | Protection | Education | Education | 0 |
| Richardson et al., 2007 | 1016 | North America (Canada) | Peace | Contextual | Risk | Impact of functioning | Current stress | 0,069 |
| Richardson et al., 2007 | 1016 | North America (Canada) | Peace | Contextual | Risk | Previous deployment experience | Number of deployments | 0,184 |
| Richardson et al., 2007 | 1016 | North America (Canada) | Peace | Contextual | Risk | Peacekeeping Stressors | Stressors during or after deployment | 0,064 |
| Richardson et al., 2008 | 25 | North America (Canada) | Peace | Individual | Risk | Impact of functioning | Mental health impairment - lack of Vitality | 0,261 |
| Richardson et al., 2008 | 25 | North America (Canada) | Peace | Individual | Risk | Impact of functioning | Mental health impairment - lack of Social functioning | 0,252 |
| Richardson et al., 2008 | 25 | North America (Canada) | Peace | Individual | Risk | Impact of functioning | Mental health impairment - Emotional role | 0,256 |
| Richardson et al., 2008 | 25 | North America (Canada) | Peace | Individual | Risk | Impact of functioning | Mental health (problems) | 0,33 |
| Richardson et al., 2008 | 25 | North America (Canada) | Peace | Individual | Risk | Impact of functioning | MSC scores (mental component summary score) | 0,7 |
| Richardson et al., 2008 | 25 | North America (Canada) | Peace | Individual | Risk | Impact of functioning | Physical role | 0,187 |
| Richardson et al., 2008 | 25 | North America (Canada) | Peace | Individual | Risk | Post-deployment psychopathology | Post-deployment depression | 0,76 |
| Richardson et al., 2009 | 707 | North America (Canada) | War | Individual | Risk | Physical health problems | Gastrointestinal disorders | 0,008 |
| Richardson et al., 2009 | 707 | North America (Canada) | War | Individual | Risk | Physical health problems | Musculoskeletal problems | 0,259 |
| Richardson et al., 2009 | 707 | North America (Canada) | War | Individual | Risk | Physical health problems | Headaches | -0,4 |
| Seedat et al., 2003 | 198 | Others (South Africa) | NA | Individual | Risk | Impact of functioning | Impairment | 0,141 |
| Seedat et al., 2003 | 198 | Others (South Africa) | NA | Individual | Risk | Post-deployment psychopathology | Post-deployment depression | 0,16 |
| Thoresen e Melhum, 2008 | 1172 | Europe (Norway) | Peace | Individual | Risk | Post-deployment psychopathology | Suicidal ideation | 0,036 |
| Yarvis & Schiess, 2008 | 1968 | North America (Canada) | NA | Individual | Risk | Physical health problems | Health problems | 0,333 |
| Yarvis & Schiess, 2008 | 1968 | North America (Canada) | NA | Individual | Risk | Post-deployment psychopathology | Post-deployment depression | 0,717 |
| Yarvis & Schiess, 2008 | 1968 | North America (Canada) | NA | Individual | Risk | Post-deployment psychopathology | Post-deployment Alcohol Misuse | 0,181 |
| NA: Not available | | | | | | | | |

# Appendix D: Coding Scheme

Bibliographical information

1. StudyID (= unique number for each study)
2. Authors of study
3. Year of publication
4. Title of study
5. Reference in APA style

Sample characteristics

1. Sample size
2. Mean age
3. Context of deployment
4. Women deployed (%)
5. PTSD incidence (%)

Study characteristics

1. Country in which the study was conducted
2. Design of the study (cross-sectional / longitudinal)
3. Time for screening
4. Instrument for screening PTSD

Variables

1. Individual / Contextual
2. Risk / Protection
3. Category
4. Factor assessed
5. Pearson product-moment correlation coefficient (*r*)

# Appendix E: List of articles that were not identified in previous reviews

1. Álvares et al., 2020
2. Dickstein et al., 2010
3. Dirkzwager et al., 2005b
4. Elhai et al., 2011
5. Forbes et al., 2016
6. Loscalzo et al., 2018
7. Orme & Kehoe, 2014b
8. Platania et al., 2020
9. Roemer et al., 1998
10. Seedat et al., 2003
11. Yarvis & Schiess, 2008

**References (Supplemental Material):**

1. Adler, A. B., Huffman, A. H., Bliese, P. D., & Castro, C. A. (2005). The Impact of Deployment Length and Experience on the Well-Being of Male and Female Soldiers. *Journal of Occupational Health Psychology*, 10(2), 121–137. <https://doi.org/10.1037/1076-8998.10.2.121>
2. Álvares, R. S. R., Mendonça-de-Souza, A. C. F., Duarte, A. F. A., Gameiro, T. M., Fischer, N. L., Souza, W. F., Coutinho, E. da S. F., Figueira, I., Volchan, E., & Souza, G. G. L. (2020). Exposure to stressful events during a peacekeeping mission may have a price: The impact on trait of negative and positive affect and mental health. *Journal of Health Psychology*, 25(9), 1285–1291. https://doi.org/10.1177/1359105317753715
3. Andrews, G., & Peters, L. (1998). The psychometric properties of the composite international diagnostic interview. *Social psychiatry and psychiatric epidemiology*, 33, 80-88.
4. Asmundson, G. G, Wright, K. D., McCreary, D.R., & Pedlar, D. (2003). Post-Traumatic Stress Disorder Symptoms in United Nations Peacekeepers: An Examination of Factor Structure in Peacekeepers with and without Chronic Pain. *Cognitive Behaviour Therapy*, 32:1, 26-37, DOI: 10.1080/16506070310003648
5. Asmundson, G. J. G., Stein, M. B., & McCreary, D. R. (2002). Posttraumatic stress disorder symptoms influence health status of deployed peacekeepers and nondeployed military personnel. *Journal of Nervous and Mental Disease*, 190(12), 807–815. https://doi.org/10.1097/00005053-200212000-00002
6. Barnes, J. B., Nickerson, A., Adler, A. B., & Litz, B. T. (2013). Perceived military organizational support and peacekeeper distress: A longitudinal investigation. *Psychological Services*, 10(2), 177–185. https://doi.org/10.1037/a0032607
7. Bartone, P. T., Vaitkus, M. A., & Adler, A. B. (1994, June). *Measuring post-traumatic stress symptoms in soldiers*. Paper presented at the United States Army Europe/Seventh Army Medical Department Symposium, GarmischPartenkirchen, Germany.
8. Blake, D., Weathers, F., Nagy, L., Kaloupek, D., Klauminzer, G., Charney, D., & Keane, T. (1990). Clinician-administered PTSD scale (CAPS). *Boston (Mass)*, 7.
9. Blake, D. D., Weathers, F. W., Nagy, L. M., Kaloupek, D. G., Gusman, F. D., Charney, D. S., & Keane, T. M. (1995). The development of a clinician-administered PTSD scale. *Journal of traumatic stress*, 8, 75-90.
10. Blake, D. D., Weathers, F. W., Nagy, L. M., Kaloupek, D. G., Klauminzer, G., Charney, D. S., & Keane, T. M. (1990). A clinician rating scale for assessing current and lifetime PTSD: The CAPS-1. *Behav Ther*; 13: 187-8.
11. Blanchard, E. B., Jones-Alexander, J., Buckley, T. C., & Forneris, C. A. (1996). Psychometric properties of the PTSD Checklist (PCL). *Behaviour research and therapy*, 34(8), 669-673.
12. Bolton, E. E., Glenn, D. M., Orsillo, S., Roemer, L., & Litz, B. T. (2003). The Relationship Between Self-Disclosure and Symptoms of Posttraumatic Stress Disorder in Peacekeepers Deployed to Somalia. *Journal of Traumatic Stress*, 16(3), 203. https://doi.org/10.1023/A:1023754820991
13. Bolton, E. E., Gray, M. J., & Litz, B. T. (2006). A cross-lagged analysis of the relationship between symptoms of PTSD and retrospective reports of exposure. *Journal of Anxiety Disorders*, 20(7), 877–895. https://doi.org/10.1016/j.janxdis.2006.01.009
14. Bolton, E. E., Litz, B. T., Britt, T. W., Adler, A., & Roemer, L. (2001). Reports of Prior Exposure to Potentially Traumatic Events and PTSD in Troops Poised for Deployment. *Journal of Traumatic Stress*, 14(1), 249. https://doi.org/10.1023/A:1007864305207
15. Bolton, E. E., Litz, B. T., Glenn, D. M., Orsillo, S., & Roemer, L. (2002). The Impact of Homecoming Reception on the Adaptation of Peacekeepers Following Deployment. *Military Psychology*, 14(3), 241–251. https://doi.org/10.1207/S15327876MP1403_4
16. Bramsen, I., Dirkzwager, A. J. E., & van der Ploeg, H. M. (2000). Predeployment personality traits and exposure to trauma as predictors of posttraumatic stress symptoms: A prospective study of former peacekeepers. *The American Journal of Psychiatry*, 157(7), 1115–1119. https://doi.org/10.1176/appi.ajp.157.7.1115
17. Castro, C. A., & Adler, A. B. (1999). OPTEMPO: Effects on soldier and unit readiness. *The US Army War College Quarterly: Parameters*, 29(3), 4.
18. Castro, C. A., Adler, A. B., & Huffman, A. H. (1999). Psychological screening of U.S. peacekeepers in Bosnia. In *Proceedings of the 41st Annual Conference of the International Military Testing Association, Monterey, California* (pp. 33–39). Retrieved from http://www .internationalmta.org/1999/99IMTAproceedings.pdf
19. Connorton, E., Perry, M. J., Hemenway, D., & Miller, M. (2011). Occupational trauma and mental illness - combat, peacekeeping, or relief work and the national co-morbidity survey replication. *Journal of Occupational and Environmental Medicine*, 53(12), 1360–1363. https://doi.org/10.1097/JOM.0b013e318234e2ec
20. Davidson, J. R., Book, S. W., Colket, J. T., Tupler, L. A., Roth, S., David, D., ... & Feldman, M. E. (1997). Assessment of a new self-rating scale for post-traumatic stress disorder.*Psychological medicine*, 27(1), 153-160.
21. Derogatis, L. R., & Melisaratos, N. (1983). The brief symptom inventory: an introductory report. *Psychological medicine*, 13(3), 595-605.
22. Di Nicola, M., Occhiolini, L., Di Nicola, L., Vellante, P., Di Mascio, R., Guizzardi, M., Colagrande, V., & Ballone, E. (2007). Stress management and factors related to the deployment of Italian peacekeepers in Afghanistan. *Military Medicine*, 172(2), 140–146. https://doi.org/10.7205/MILMED.172.2.140
23. Dickstein, B. D., Suvak, M., Litz, B. T., & Adler, A. B. (2010). Heterogeneity in the course of posttraumatic stress disorder: Trajectories of symptomatology. *Journal of Traumatic Stress*, 23(3), 331–339. https://doi.org/10.1002/jts.20523
24. Dirkzwager, A. J. ., Bramsen, I., & van der Ploeg, H. M. (2003). Social support, coping, life events, and posttraumatic stress symptoms among former peacekeepers: a prospective study. *Personality and Individual Differences*, 34(8), 1545–1559. https://doi.org/10.1016/S0191-8869(02)00198-8
25. Dirkzwager, A. J. E., Bramsen, I., & Van Der Ploeg, H. M. (2005a). Factors associated with posttraumatic stress among peacekeeping soldiers. *Anxiety, Stress & Coping*, 18(1), 37–51. https://doi.org/10.1080/10615800412336418
26. Dirkzwager, A. J. E., Bramsen, I., Adèr, H., & van der Ploeg, H. M. (2005b). Secondary Traumatization in Partners and Parents of Dutch Peacekeeping Soldiers. *Journal of Family Psychology*, 19(2), 217–226. https://doi.org/10.1037/0893-3200.19.2.217
27. Elhai, J. D., Richardson, J. D., & Pedlar, D. J. (2007). Predictors of general medical and psychological treatment use among a national sample of peacekeeping veterans with health problems. *Journal of Anxiety Disorders*, 21(4), 580-589.
28. Engdahl, R. M., Elhai, J. D., Richardson, J. D., & Frueh, B. C. (2011). Comparing Posttraumatic Stress Disorder’s Symptom Structure between Deployed and Nondeployed Veterans. *Psychological Assessment*, 23(1), 1–6.
29. Forbes, D., Bennett, N., Biddle, D., Crompton, D., McHugh, T., Elliott, P., & Creamer, M. (2005). Clinical Presentations and Treatment Outcomes of Peacekeeper Veterans With PTSD: Preliminary Findings. *The American Journal of Psychiatry*, 162(11), 2188–2190. https://doi.org/10.1176/appi.ajp.162.11.2188
30. Forbes, D., O’Donnell, M., Brand, R. M., Korn, S., Creamer, M., McFarlane, A. C., Sim, M. R., Forbes, A. B., & Hawthorne, G. (2016). The long-term mental health impact of peacekeeping: prevalence and predictors of psychiatric disorder. *BJPsych Open*, 2(1), 32–37. https://doi.org/10.1192/bjpo.bp.115.001321
31. Forbes, D., Creamer, M., & Biddle, D. (2001). The validity of the PTSD checklist as a measure of symptomatic change in combat-related PTSD. *Behaviour research and therapy*, 39(8), 977-986.
32. Geuze, E., Vermetten, E., de Kloet, C. S., Hijman, R., & Westenberg, H. G. M. (2009). Neuropsychological performance is related to current social and occupational functioning in veterans with posttraumatic stress disorder. *Depression & Anxiety* (1091-4269), 26(1), 7–15. https://doi.org/10.1002/da.20476
33. Gjerstad, C. L., Bøe, H. J., Falkum, E., Martinsen, E. W., Nordstrand, A. E., Tønnesen, A., Reichelt, J. G., & Lystad, J. U. (2020b). Prevalence and Correlates of Mental Health Problems in Norwegian Peacekeepers 18–38 Years Postdeployment. *Journal of Traumatic Stress*, 33(5), 762–772. https://doi.org/10.1002/jts.22578
34. Goldberg, D. P., & Williams, P. (1988). *A user's guide to the General Health Questionnaire*.
35. Gori, A., Giannini, M., and Schuldberg, D. (2015). PTI – Psychological Treatment Inventory. *Manual*. Florence: Giunti OS-Organizzazioni Speciali.
36. Gravel, R., & Béland, Y. (2005). The Canadian Community Health Survey: mental health and well-being. *The Canadian Journal of Psychiatry*, 50(10), 573-579.
37. Gray, M. J., Bolton, E. E., & Litz, B. T. (2004). A Longitudinal Analysis of PTSD Symptom Course: Delayed-Onset PTSD in Somalia Peacekeepers. *Journal of Consulting and Clinical Psychology*, 72(5), 909–913. https://doi.org/10.1037/0022-006X.72.5.909
38. Greenberg, N., Iversen, A., Hull, L., Bland, D., & Wessely, S. (2008). Getting a peace of the action: measures of post traumatic stress in UK military peacekeepers. *Journal of the Royal Society of Medicine*, 101(2), 78–84. <https://doi.org/10.1258/jrsm.2007.070024>
39. Horowitz, M., Wilner, N., & Alvarez, W. (1979). Impact of Event Scale: A measure of subjective stress. *Psychosomatic medicine*, 41(3), 209-218.
40. Hotopf, M., David, A., Hull, L., Ismail, K., Unwin, C., & Wessely, S. (2003). The Health Effects of Peacekeeping (Bosnia, 1992-1996): A Cross-Sectional Study - Comparison with Nondeployed Military Personnel. *Military Medicine*, 168(5), 408–413.
41. Hovens, J. E., Van der Ploeg, H. M., Klaarenbeek, M. T. A., Bramsen, I., Schreuder, J. N., & Rivero, V. V. (1994). The assessment of posttraumatic stress disorder: with the Clinician Administered PTSD Scale: Dutch results. *Journal of clinical psychology*, 50(3), 325-340.
42. Hovens, J. E., Bramsen, I., Van der Ploeg, H. M., & Reuling, I. E. W. (2000). Test-retest reliability of the trauma and life events self-report inventory. *Psychological reports*, 87(3), 750-752.
43. Keane, T. M., Caddell, J. M., & Taylor, K. L. (1988). Mississippi Scale for Combat-Related Posttraumatic Stress Disorder: three studies in reliability and validity. *Journal of consulting and clinical psychology*, 56(1), 85.
44. Kessler, R. C., & Üstün, T. B. (2004). The world mental health (WMH) survey initiative version of the world health organization (WHO) composite international diagnostic interview (CIDI). *International journal of methods in psychiatric research*, 13(2), 93-121.
45. Kessler, R. C., Abelson, J., Demler, O., Escobar, J. I., Gibbon, M., Guyer, M. E., ... & Zheng, H. (2004). Clinical calibration of DSM‐IV diagnoses in the World Mental Health (WMH) version of the World Health Organization (WHO) Composite International Diagnostic Interview (WMH‐CIDI). *International journal of methods in psychiatric research*, 13(2), 122-139.
46. Klaassens, E. R., van Veen, T., Weerts, J. M. P., & Zitman, F. G. (2008). Mental health of Dutch Peacekeeping Veterans 10–25 years after deployment. *European Psychiatry*, 23(7), 486–490. https://doi.org/10.1016/j.eurpsy.2008.03.009
47. Litz, B., Orsillo, S., Friedman, M., Ehlich, P. & Batres, A. (1997a). Posttraumatic stress disorder associated with peacekeeping duty in Somalia for U.S. military personnel. *The American journal of psychiatry*. 154. 178-84. DOI: 10.1176/ajp.154.2.178.
48. Litz, B. T., King, L. A., King, D. W., Orsillo, S. M., & Friedman, M. J. (1997b). Warriors as peacekeepers: Features of the Somalia experience and PTSD. *Journal of Consulting and Clinical Psychology*, 65(6), 1001–1010. https://doi.org/10.1037/0022-006X.65.6.1001
49. Loscalzo, Y., Giannini, M., Gori, A., & Di Fabio, A. (2018). The Wellbeing of Italian Peacekeeper Military: Psychological Resources, Quality of Life and Internalizing Symptoms. *Frontiers in Psychology*, 9. https://doi.org/10.3389/fpsyg.2018.00103
50. Maguen, S., Litz, B. T., Wang, J. L., & Cook, M. (2004). The Stressors and Demands of Peacekeeping in Kosovo: Predictors of Mental Health Response. *Military Medicine*, 169(3), 198–206. https://doi.org/10.7205/MILMED.169.3.198
51. Maguen, S., Stalnaker, M., McCaslin, S., & Litz, B. T. (2009). PTSD Subclusters and Functional Impairment in Kosovo Peacekeepers. *Military Medicine*, 174(8), 779–785. https://doi.org/10.7205/MILMED-D-03-2808
52. Mehlum, L., & Weisæth, L. (2002). Predictors of Posttraumatic Stress Reactions in Norwegian U.N. Peacekeepers 7 Years After Service. *Journal of Traumatic Stress*, 15(1), 17. https://doi.org/10.1023/A:1014375026332
53. Mehlum, L., Koldsland, B. O., & Loeb, M. E. (2006). Risk Factors for Long-Term Posttraumatic Stress Reactions in Unarmed UN Military Observers: A Four-Year Follow-Up Study. *Journal of Nervous and Mental Disease*, 194(10), 800–804. https://doi.org/10.1097/01.nmd.0000240189.20531.2d
54. Orme, G. J., & Kehoe, E. J. (2014a). Hardiness as a predictor of mental health and well-being of Australian Army reservists on and after stability operations. *Military Medicine*, 179(4), 404–412. https://doi.org/10.7205/MILMED-D-13-00390
55. Orme, G. J., & Kehoe, J. (2014b). Reservists in a Postconflict Zone: Deployment Stressors and the Deployment Experience. *Military Medicine*, 179(2), 137–142. https://doi.org/10.7205/MILMED-D-13-00439
56. Platania, S., Castellano, S., Petralia, M.C., Digrandi, F., Coco, M., Pizzo, M., Di Nuovo, S. (2020). The moderating effect of the dispositional resilience on the relationship between Post-traumatic Stress Disorder and the professional quality of life of the military returning from the peacekeeping operations. *Mediterranean Journal of Clinical Psychology*, 8 (3), pp. 1-21. 10.6092/2282-1619/mjcp-2560
57. Rademaker, A., Kleber, R., Meijer, M., & Vermetten, E. (2009). Investigating the MMPI-2 Trauma Profile in Treatment-Seeking Peacekeepers. *Journal of Personality Assessment*, 91(6), 593–600. https://doi.org/10.1080/00223890903230899
58. Raphael B, Lundin T, Weisaeth L (1989). A research method for the study of psychological and psychiatric aspects of disaster. *Acta Psychiatr Scand Suppl*. 353:1–75.
59. Richardson, J. D., Long, M. E., Pedlar, D., & Elhai, J. D. (2008). Posttraumatic stress disorder and health-related quality of life among a sample of treatment- and pension-seeking deployed Canadian forces peacekeeping veterans. *The Canadian Journal of Psychiatry / La Revue Canadienne de Psychiatrie*, 53(9), 594–600. https://doi.org/10.1177/070674370805300906
60. Richardson, J. D., Naifeh, J. A., & Elhai, J. D. (2007). Posttraumatic stress disorder and associated risk factors in Canadian peacekeeping veterans with health-related disabilities. *The Canadian Journal of Psychiatry / La Revue Canadienne de Psychiatrie*, 52(8), 510–518. https://doi.org/10.1177/070674370705200809
61. Richardson, J. D., Pekevski, J., & Elhai, J. D. (2009). Post-traumatic stress disorder and health problems among medically ill Canadian peacekeeping veterans. *Australian & New Zealand Journal of Psychiatry*, 43(4), 366–372. https://doi.org/10.1080/00048670902721061
62. Roemer, L., Litz, B. T., Orsillo, S. M., Ehtich, P. J., & Friedman, M. J. (1998). Increases in Retrospective Accounts of War-Zone Exposure Over Time: The Role of PTSD Symptom Severity. *Journal of Traumatic Stress*, 11(3), 597–605. https://doi.org/10.1023/A:1024469116047
63. Sareen, J., Belik, S.-L., Afifi, T. O., Asmundson, G. J. G., Cox, B. J., & Stein, M. B. (2008). Canadian Military Personnel’s Population Attributable Fractions of Mental Disorders and Mental Health Service Use Associated With Combat and Peacekeeping Operations. *American Journal of Public Health*, 98(12), 2191–2198. https://doi.org/10.2105/AJPH.2008.134205
64. Sareen, J., Cox, B. J., Afifi, T. O., Stein, M. B., Belik, S.-L., Meadows, G., & Asmundson, G. J. G. (2007). Combat and peacekeeping operations in relation to prevalence of mental disorders and perceived need for mental health care: Findings from a large representative sample of military personnel. *Archives of General Psychiatry*, 64(7), 843–852. https://doi.org/10.1001/archpsyc.64.7.843
65. Sareen, J., Henriksen, C. A., Bolton, S.-L., Afifi, T. O., Stein, M. B., & Asmundson, G. J. G. (2013). Adverse childhood experiences in relation to mood and anxiety disorders in a population-based sample of active military personnel. *Psychological Medicine*, 43(1), 73–84. https://doi.org/10.1017/S003329171200102X
66. Seedat, S., le Roux, C., & Stein, D. J. (2003). Prevalence and Characteristics of Trauma and Post-Traumatic Stress Symptoms in Operational Members of the South African National Defence Force. *Military Medicine*, 168(1), 71–75.
67. Souza, W. F., Figueira, I., Mendlowicz, M. V., Volchan, E., Mendonça-de-Souza, A. C., Duarte, A. F. A., da Silva, Â. M. M., Marques-Portella, C., Mari, J. J., & Coutinho, E. S. F. (2008). Negative affect predicts posttraumatic stress symptoms in Brazilian volunteer United Nations peacekeepers in Haiti. *Journal of Nervous and Mental Disease*, 196(11), 852–855. https://doi.org/10.1097/NMD.0b013e31818b4682
68. Stapleton, J. A., Asmundson, G. J. G., Woods, M., Taylor, S., & Stein, M. B. (2006). Health Care Utilization by United Nations Peacekeeping Veterans with Co-occurring, Self-Reported, Post-Traumatic Stress Disorder and Depression Symptoms versus Those Without. *Military Medicine*, 171(6), 562–566. https://doi.org/10.7205/MILMED.171.6.562
69. Thoresen, S., & Mehlum, L. (2008). Traumatic stress and suicidal ideation in Norwegian male peacekeepers. *Journal of Nervous and Mental Disease*, 196(11), 814–821. https://doi.org/10.1097/NMD.0b013e31818b6a92
70. U.N. (1995). *Department of Peacekeeping Operations: U.N. Stress Management Booklet*. New York, NY. U.N. Department of Peacekeeping Operations.
71. Vandenbroucke, J. P., Elm, E. V., Altman, D. G., Gøtzsche, P. C., Mulrow, C. D., Pocock, S. J., ... & Strobe Initiative. (2007). Strengthening the Reporting of Observational Studies in Epidemiology (STROBE): explanation and elaboration. *Annals of internal medicine*, 147(8), W-163.
72. Waller M., Treloar S. A., Sim M. R., McFarlane A. C., McGuire A. C. L., Bleier J., & Dobson A. J. (2012). Traumatic events, other operational stressors and physical and mental health reported by Australian Defence Force personnel following peacekeeping and war-like deployments. *BMC Psychiatry*, 12(1), 88. https://doi.org/10.1186/1471-244X-12-88
73. Ward, W. (1997). Psychiatric morbidity in Australian veterans of the United Nations peacekeeping force in Somalia. *Australian and New Zealand Journal of Psychiatry*, 31(2), 184–193. https://doi.org/10.3109/00048679709073819
74. Weathers, F. W., Litz, B. T., Herman, D. S., Huska, J. A., & Keane, T. M. (1993, October). The PTSD Checklist (PCL): Reliability, validity, and diagnostic utility. In *Annual Meeting of the International Society for Traumatic Stress Studies, San Antonio, TX*, October, 1993.
75. Weathers, F. W. F. J., & Ford, J. (1996). Psychometric review of PTSD Checklist (Pcl-C, Pcl-S, Pcl-M, Pcl-Pr). *Measurement of stress, trauma, and adaptation*, 1, 250-251.
76. Weathers, F. W., Keane, T. M., & Davidson, J. R. (2001). Clinician‐Administered PTSD Scale: A review of the first ten years of research. *Depression and anxiety*, 13(3), 132-156.
77. Weathers, F. W., Huska, J. A., & Keane, T. M. (1991). PCL-C for DSM-IV. Boston, MA: Behavioral Science Division. *National Center for PTSD*.
78. Yarvis, J. S., & Schiess, L. (2008). Subthreshold Posttraumatic Stress Disorder (PTSD) as a Predictor of Depression, Alcohol Use, and Health Problems in Veterans. *Journal of Workplace Behavioral Health*, 23(4), 395–424. https://doi.org/10.1080/15555240802547801
